# An immunochemistry-based screen for chemical inhibitors of DNA-protein interactions and its application to human CGGBP1

**DOI:** 10.1186/s12885-020-07526-5

**Published:** 2020-10-20

**Authors:** Manthan Patel, Divyesh Patel, Subhamoy Datta, Umashankar Singh

**Affiliations:** HoMeCell Lab, Biological Engineering, Indian Institute of Technology Gandhinagar, Gandhinagar, Gujarat 382355 India

**Keywords:** DNA-protein interaction, Library screen, CGGBP1, Givinostat

## Abstract

**Background:**

Inhibition of DNA-binding of proteins by small-molecule chemicals holds immense potential in manipulating the activities of DNA-binding proteins. Such a chemical inhibition of DNA-binding of proteins can be used to modulate processes such as replication, transcription, DNA repair and maintenance of epigenetic states. This prospect is currently challenged with the absence of robust and generic protocols to identify DNA-protein interactions. Additionally, much of the current approaches to designing inhibitors requires structural information of the target proteins.

**Methods:**

We have developed a simple dot blot and immunodetection-based assay to screen chemical libraries for inhibitors of DNA-protein interactions. The assay has been applied to a library of 1685 FDA-approved chemicals to discover inhibitors of CGGBP1, a multifunctional DNA-binding protein with no known structure. Additional in vitro and *in cellulo* assays have been performed to verify and supplement the findings of the screen.

**Results:**

Our primary screen has identified multiple inhibitors of direct or indirect interactions between CGGBP1 and genomic DNA. Of these, one inhibitor, Givinostat, was found to inhibit direct DNA-binding of CGGBP1 in the secondary screen using purified recombinant protein as the target. DNA and chromatin immunoprecipitation assays reinforced the findings of the screen that Givinostat inhibits CGGBP1-DNA binding.

**Conclusions:**

The assay we have described successfully identifies verifiable inhibitors of DNA-binding of protein; in this example, the human CGGBP1. This assay is customizable for a wide range of targets for which primary antibodies are available. It works with different sources of the target protein, cell lysates or purified recombinant preparations and does not require special equipment, DNA modifications or protein structural data. This assay is scalable and highly adaptable with the potential to discover inhibitors of transcription factors with implications in cancer biology.

## Background

Chemical inhibition of protein activities has been largely limited to enzymatic proteins [1–3]. Structure-aided drug design has been a standard approach towards designing novel inhibitors against new targets [4, 5]. In the last decades, the advent of large scale screening technologies has allowed researchers to screen libraries of small molecule chemicals for their inhibitory activities against chosen target enzymes [6–8]. The latter is a blind hit and trial approach, which can help us identify sub-optimal inhibitors of a target enzyme from a library that would need further optimization. Such optimization could be driven by the structural information of the target protein or for reducing the concentration of the chemical required for efficient inhibition. The IC_50_ values for small-molecule bioactive chemicals are centered on such a model wherein the chemicals inhibit an enzyme by occupying a defined binding site; often a single pocket per protein molecule [8]. The large scale inhibition screens for enzymatic activities have been used exhaustively and yet, for many inhibitor-target combinations, we do not have sufficient information about specificity, secondary targets and concentration-dependent changes in target profiles for any given inhibitor [9–11]. The DNA-binding proteins, some of which bind to specific sequences and others to a wide range of sequences or sequence independently, present a challenge as potentially useful but difficult drug targets [12]. DNA-protein interactions do not conform to the same biochemical principles as applied to the substrate-enzyme combinations. Thus, the knowledge and strategies of enzyme inhibitions by chemicals can not be simply extended for inhibition of DNA-protein interactions.

DNA-binding of proteins is central to cell biology [13]. Mitigation of the activities of DNA-binding proteins has been classically limited to mutations in the DNA-binding domains that would impair the interaction between DNA and the mutant protein expressed by a transgene [14–16]. Alternatively, other mutations could interfere with the indirect associations between a protein of interest and chromatin [17]. More recently, RNA interference has been used to post-transcriptionally silence DNA-binding proteins to study the effects of their functional inhibition [18]. CRISPR-Cas9 has further enabled us to use mutagenesis as a means to inhibit the DNA-binding due to targeted mutations in selected regions of a protein of interest [19, 20]. There is a paucity of methods to identify chemical inhibitors of DNA-binding of proteins [21]. Chemical inhibition of DNA-binding of proteins would open up several experimental possibilities in cancer biology. Unlike the above-mentioned techniques involving mutations (that is, stable and irreversible) or RNA interference (that is, slow and transitory), chemical inhibition of DNA-binding would allow rapid targeting of proteins such as transcription factors, chromatin modulators, and regulators of replication and DNA repair.

Methods to identify chemical inhibitors or facilitators of DNA-binding have been proposed recently [21–23]. The method described by Alonso et al. [21] requires synthesis of biotinylated DNA oligonucleotides to be used as DNA targets in the assay. This ELISA-based protocol does not differentiate between inhibitors that inhibit the binding by either occupying and blocking the DNA from those which might bind to the target protein as well. Thus the method identifies compounds that bind to target DNA sequences and thus obstruct the binding of proteins to those cognate sequences. The usage of biotinylated short oligonucleotides renders this method expensive and difficult for screening inhibitors of proteins that (i) may not bind to short defined DNA sequence motifs or (ii) require unpredictable DNA features for binding, such as the formation of DNA secondary structures and presence of repetitive sequences. This technique could be applied only for a panel of 29 compounds against HMGA2 [21]. However, the advantage of this technique was that it could be used to identify direct inhibitors of binding sites of candidate proteins. Inhibition of indirect binding to DNA could be affected by chemicals that interfere with protein-protein binding wherein one of the two proteins is a DNA-binding protein. One such efficient screen for approximately 75,000 chemicals was described for inhibition of interactions between FANCM and RMI [24]. The proximity-based method used inhibitors to disrupt preformed complexes. Despite the advantages of this method, it could not be used for identifying direct inhibitors of DNA-protein binding [24].

A photonic crystal biosensor-based method has also been described [22]. This method presents some advantages compared to the other methods mentioned above. It can be applied to sequence-specific DNA-binding proteins as well as sequence-independent DNA-binding proteins. The authors applied it successfully to two candidate protein targets: MazEF (sequence-specific binder) and AIF (sequence-independent) using a library of 1000 compounds. The concentrations of some putative inhibitors used for screening against AIF in this assay ranged from 25 μM to 200 μM, whereas, the IC_50_ range was from 23 μM to 50 μM for ATA, an inhibitor positively identified for AIF. This method, however, also requires the usage of biotinylated DNA and custom fabrication of optical sensors thereby increasing the costs and customizability [22]. In yet another study an in vitro inhibition of DNA Ligase IV by SCR7 was achieved by using a concentration of 200 μM [25].

Here we present a method, called DBID, for screening small molecule chemical libraries with low cost and no specialized equipment requirements. This method does not require biotinylated DNA oligonucleotides and is blind to the nature of DNA that an experimenter wants to test as a potential binding site for any DNA-binding protein. It is easily customizable for any protein-antibody-DNA combination and can be used at a common library-wide chemical concentration choice for the screening. We demonstrate this method here using a library of 1685 chemicals against a human protein called CGGBP1.

The human CGGBP1, a 20 KDa protein, was discovered as a CGG triplet repeat-binding factor [26]. The human protein atlas and the cancer genome atlas describe the widespread expression of CGGBP1 mRNA and protein in normal cells as well as a variety of cancers [27, 28]. Earlier, CGGBP1 was described as a transcriptional repressor with cytosine methylation-sensitive binding at FMR1 promoter [29]. More recently, a series of investigations have shown that CGGBP1 is a multifunctional protein [30–32]. It regulates CDKN1A, TP53 [33] levels and is required for normal cytokinesis and prevention of tetraploidization [34]. CGGBP1 expression in cancer cells is responsive to stress and depletion of CGGBP1 leads to a stress response-like gene expression pattern [32]. Interestingly, a variety of cancer cell types exhibit a dependence on CGGBP1 that resembles the non-oncogene addiction phenomenon described for heat shock stress and proteotoxic stress response genes [35], some of which are regulated by CGGBP1 [31, 33, 36]. This seems to involve the derepression of repetitive sequences, mostly the CGGBP1 binding LINEs and SINEs thereby leading to anomalies in RNA Pol-II activities [37–41].

The differential dependence of normal and cancer cells on CGGBP1 suggests that inhibiting it may be a useful means to target cancer cells. A small-compound inhibition of functions of CGGBP1 would be a valuable tool in cancer biology research.

By applying our screening method we have identified that the HDAC inhibitor anti-cancer agent [42–47] Givinostat also inhibits CGGBP1-DNA binding. We have also verified this inhibition of CGGBP1-DNA binding inhibition by Givinostat using in vitro and *in cellulo* assays, including DBID assays on histones. Our results show that the DBID assay can be used to screen chemical libraries for DNA-binding proteins. The possibility of chemical inhibition of DNA-binding protein holds immense potential in cancer biology research.

## Methods

### Cell culture and cell lysate preparation

HEK293T cells were grown in DMEM (AL007A, Himedia) supplemented with 10% FBS (RM1112, Himedia) and antibiotic-antimycotic agent (15,240,062, Gibco). Cells were collected and washed with PBS (TL1006, Himedia). Washed cell pellet was lysed in RIPA buffer (150 mM NaCl, 5 mM EDTA, 50 mM Tris, 1% NP40 (IGEPAL), 0.1% Na-deoxycholate and 0.1% SDS) containing Halt protease and phosphatase inhibitor cocktail (PI78441, Invitrogen).

### Crosslinking of DNA with membrane disc

Genomic DNA was isolated from HEK293T cells [48]. DNA was sonicated using a Diagenode Bioruptor sonicator for 21 cycles at 30 s “ON” followed by 30 s “OFF”. Sonication was standardised to get ~ 1 kb fragment size DNA. Sonicated genomic DNA was dissolved in 1x TE buffer at 200 ng/μl. Nylon-membrane (GX222020NN, Genetix) discs (5 mm diameter) were soaked with the sonicated DNA overnight and were crosslinked by vacuum heating the wet membranes at 80 °C for 90 min. DNA crosslinked nylon-membrane discs (called dot blots) were transferred to 96 well plates and incubated with blocking solution (PBST with 10% FBS). For the primary screen, one dot blot was used for each of the 1685 compounds (L1100, Selleckchem) distributed across multiple 96 well plates. In addition, the control samples, as described below, were run on two dot blots for each 96 well plate.

### Dot-blot assay

DNA-crosslinked nylon membranes were pre-incubated with blocking solution for 90 min. Cell lysate (15 μl of a 25 ml lysate stock obtained from approximately 500 million cells) was incubated with compounds (pre-dissolved in DMSO and diluted to a final concentration of 100 μM) or compound diluent (0.05% DMSO in 1x PBS) for 30 min. The concentration of 100 μM for the chemicals was selected with the following considerations: The C_max_ of cancer chemotherapeutic agents (mostly inhibitors of enzymatic activities) has a wide range (35.04 ± 145.66 μM) with a median of 1.18 μM. The median C_max_ of 1 μM for approximately 0.5-1 million cells in 10 ml of culture medium translates into 20-10 nmoles of chemical per 1 million cells. We extracted 500 million cells into 25 ml lysate and used 15 μl of this extract with 100 μM of each chemical in a 30 μl reaction volume. Thus, the 100 μM concentration translated into 20-10 nmoles of chemical for every million cells. This also approximated the median C_max_ of 1 μM. The chosen concentration was thus optimal only for the in vitro DBID assay only. Pre-incubated compound-lysate mix was transferred to DNA-crosslinked nylon membrane and incubated for 90 min at 4 °C. The dot blots were washed three times with 1x PBS and protein-DNA complexes were crosslinked with 4% formaldehyde in 1x PBS. The dot blots were washed three times with PBST and then incubated with 50 μl of rabbit polyclonal anti-CGGBP1 antibody mix (a mix of 1:120 dilutions of SC-292517, SCBT and 10,716-1-AP, Proteintech) overnight with gentle rocking. Dot blots were washed with PBST three times and incubated with 30 μl of the biotinylated anti-rabbit secondary antibody for 90 min (1:10 dilution, 865,002, R&D Systems). After three PBST washes of 5 min each, the dot blots were incubated with high sensitivity streptavidin conjugated to horseradish peroxidase (1:10 dilution, 865,006, R&D Systems) for 90 min. All antibody dilutions and streptavidin-HRP conjugate dilutions were done in the blocking solution. Dot blots were washed three times with PBST and incubated with 15 μl of DAB (3,3′-Diaminobenzidine) chromogen (2 ml of DAB chromogen (860,001, R&D Systems) diluted in DAB chromogen buffer (860,005, R&D Systems)). Positive and negative controls are described below.

**Table:** 

	Cell lysate	Primary antibody	Secondary antibody (biotinylated anti-rabbit IgG)
Positive control	Yes	Anti-CGGBP1 rabbit polyclonal IgG	Yes
Negative control 1	No	Anti-CGGBP1 rabbit polyclonal IgG	Yes
Negative control 2	Yes	No	Yes
Negative control 3	Yes	IgG	Yes
Negative control 4	Yes	Anti-CGGBP1 rabbit polyclonal IgG	No

### Secondary screening for direct or indirect inhibition of the primary hits

The secondary screening was performed for eight inhibitors obtained as hits from the primary screen along with a positive control (no inhibitor) and a negative control (no primary antibody). The assay was done in 6-well plates with each inhibitor-rCGGBP1 combination assayed in 8-10 technical replicates. For each sample, dot blots were incubated with 250 μl of blocking solution (10% FBS in PBS) for 1 h at room temperature in a moist chamber. Simultaneously, the rCGGBP1 (500 ng per compound) diluted in RIPA lysis buffer (containing protease phosphatase inhibitor cocktail) was incubated with inhibitors (at a final concentration of 100 μM for 45 min) at 4 °C. In the positive control sample, the rCGGBP1 diluted in RIPA lysis buffer was incubated with the compound diluent (0.05% DMSO in 1x PBS). The protein-inhibitor mix was diluted in the blocking solution such that the final volume was 250 μl for each sample. The blocking solution was removed and rCGGBP1-inhibitor mix was transferred onto the membranes in each well for 1 h at 4 °C in a moist chamber with gentle rocking. The blots were washed thrice with PBS. This was followed by fixation of the interactions using 1% PFA for 5 min and subsequent PBST washes three times. Membranes were incubated with mouse anti-FLAG antibody (1:1000 of SC-166384, SCBT, in blocking solution) for 1 h at room temperature with gentle rocking, followed by washing three times with PBST. The membranes were then incubated with anti-mouse HRP-conjugated secondary antibody (1:5000 of NA931, GE Healthcare) for 1 h at room temperature followed by three washes with PBST. The signal was detected with ECL substrate (32,106, Pierce) by using Biorad ChemiDoc MP Imaging System. Further, the membranes were stripped with 0.2 N NaOH for 5 min and incubated with anti-CGGBP1 antibody (1:1000 of 10,716-1-AP, Proteintech, in blocking solution) overnight at 4 °C followed by three PBST washes. The membranes were incubated with anti-rabbit HRP-conjugated secondary antibody (1:5000 of NA934, GE Healthcare) for 1 h at room temperature and subsequently washed with PBST thrice. The signal was captured detecting chemiluminescence as described above. The signal was quantified by densitometry analysis of images (.tif format) using ImageJ software. The statistical analysis and data presentation were performed using OpenOffice and GraphPad Prism8. The DBID assay was also performed for the Givinostat-CGGBP1 combination using a lower concentration range (10 μM, 1 μM, 0.1 μM, 0.01 μM and 0.001 μM) of Givinostat. This assay was repeated to detect the inhibition of DNA-binding of total histone H3 (without fixation) and histone H3 post-translational modifications: H3K9me3 and H3K4me3 (with fixation) by Givinostat.

### Synthesis of Alu DNA

For the generation of full-length Alu DNA, an established DNA-binding target of CGGBP1, the full-length consensus sequence of Alu SINE was synthesized in five overlapping oligonucleotides. The 5′ end of the first fragment and the 3′ end of the last fragment contained the T7 and SP6 primers respectively. The Alu DNA product was obtained through overlapping PCR using an equimolar mix of overlapping oligonucleotides as template and T7 and SP6 sequences as primers. The PCR product was run on the agarose gel and purified using the PCR purification kit (A1222, Promega). The Alu PCR product was cloned into using the pGEM-T Easy Vector (A1380, Promega). The clones obtained were subjected to Sanger sequencing for verification. This clone was used as a template for amplifying Alu DNA for in vitro DNA-rCGGBP1 immunoprecipitation assay. Two different lengths of Alu DNA were amplified due to two priming sites for T7 as well as SP6 in the clone (~ 320 bp (T7 and SP6 sites in the insert) and other at ~ 400 bp (T7 and SP6 sites in the vector backbone)). The Alu DNA was amplified using either unmethylated cytosine or 5′-methylated dCTPs. The sequence of the Alu DNA is as follows:

5′-TAATACGACTCACTATAGGGGGCCGGGCGCGGTGGCTCACGCCTGTAATCCCAGCAC TTTGGGAGGCCGAGGCGGGAGGATCGCTTGAGCCCAGGAGTTCGAGACCAGCCTGGGCAACATAGCGAGACCCCGTCTCTACAAAAAATACAAAAATTAGCCGGGCGTGGTGGCGCGCGCCTGTAGTCCCAGCTACTCGGGAGGCTGAGGCAGGAGGATCGCTTGAGCCCAGGAGTTCGAGGCTGCAGTGAGCTATGATCGCGCCACTGCACTCCAGCCTGGGCGACAGAGCGAGACCCTGTCTCTTCTATAGTGTCACCTAAAT-3′

### In vitro DNA-IP and qPCR

The in vitro DNA-IP was performed with or without Givinostat. rCGGBP1 (0.5 μg) diluted in 1x PBS was incubated with an inhibitor (compound was pre-dissolved in DMSO and diluted to 100 μM final concentration) for 45 min at room temperature. The final volume was adjusted to 50 μl with PBS. The protein-inhibitor mix was incubated with 1 μg of Alu DNA. Simultaneously 3 μg of the anti-FLAG antibody (described above) was subjected to incubation with protein-G sepharose beads (60 μl, 17,061,801, GE Healthcare) for 1 h with tumbling. The DNA-protein-inhibitor mix was transferred to the tube containing the anti-FLAG antibody-bound protein-G sepharose beads and incubated for 60 min at room temperature with tumbling. The beads were allowed to settle down followed by a gentle spin and the supernatant containing the unbound antibody and DNA was removed. The beads were gently washed three times with ice-cold 1x PBS. For each sample, the 1x TE buffer (40 μl per sample) was added and mixed followed by heating at 80 °C for 20 min to elute the bound DNA.

The immunoprecipitated Alu DNA in presence and absence of Givinostat was used as a template for the qPCR (1,725,124, Biorad) using T7 and SP6 primers. The PCR was performed for both the samples (Alu DNA immunoprecipitated with Givinostat-inhibited and mock-inhibited rCGGBP1). The template was used at different dilutions of the immunoprecipitated DNA (1:100 and 1:200 diluted) for qPCR in multiple replicates. The input Alu DNA template was used as a control to calculate the first delta Ct (dCt). The second delta Ct values were calculated by subtracting the dCt values obtained for the mock-inhibited sample from those of the Givinostat-inhibited sample. Following are the PCR conditions for Alu PCR: 95 °C-5 min, (95 °C- 20 s, 55 °C- 20 s, 72 °C-30 s, 80 °C-30 s) × 50, melting curve analysis (50 °C to 95 °C with constant signal recording).

### ChIP-qPCR

CGGBP1 ChIP was performed as described earlier [41]. HEK293T cells were treated with 2 μM Givinostat or DMSO for 6 h and 24 h. Approximately 1 million cells were crosslinked (1% formaldehyde solution, 37 °C for 10 min, quenched with 125 mM Glycine) and harvested. After washing twice with PBS, the cell pellet was lysed in SDS lysis buffer supplemented with protease inhibitor cocktail (PI78441, Invitrogen). Resuspended and cleared chromatin was sonicated using a Diagenode Bioruptor (20 cycles of 30 s “ON”/30 s “OFF”). As per our previous experience, the sonication conditions were optimised for generating DNA fragments of length range 0.5 kb to 1 kb. The sonicated chromatin was centrifuged (16,000 rcf, 5 min, 4 °C) and 30 μl was set aside as input. CGGBP1 immunoprecipitation was performed on 120 μl of the cleared sonicated lysate diluted using ChIP dilution buffer (0.01% SDS, 1.1% Triton X-100, 1.2 mM EDTA, 16.7 mM Tris-HCl, pH 8.1, 167 mM NaCl) supplemented with protease inhibitor cocktail and precleared using protein G sepharose beads for 4 h at 4 °C. The anti-CGGBP1 antibody was added and tumbled overnight at 4 °C followed by protein G sepharose beads for 1 h. Protein G sepharose beads were collected by gentle centrifugation and washed with three buffers in the following order: low-salt IP wash buffer (0.1% SDS, 1% Triton X-100, 2 mM EDTA, 20 mM Tris–HCl and 150 mM NaCl), high-salt IP wash buffer (0.1% SDS, 1% Triton X-100, 2 mM EDTA, 20 mM Tris–HCl and 500 mM NaCl), LiCl IP wash buffer (0.25 M LiCl, 1% IGEPAL, 1% Na deoxycholate, 1 mM EDTA and 10 mM Tris–HCl). Finally, the Protein G sepharose beads were washed twice with TE buffer (10 mM Tris–HCl and 1 mM EDTA). The DNA bound to the Protein G sepharose beads was extracted in an elution buffer (1% SDS and 0.1 M NaHCO3), subjected to reverse crosslink (addition of 20 μl of 5 M NaCl and incubation at 65 °C for 6 h), followed by addition of 10 μl of 0.5 M EDTA pH 8.0 and 20 μl of 1 M Tris-HCl pH 6.8, and then by the addition of 2 μl of 10 mg/ml Proteinase K (P2308, Sigma) and digestion for 1 h at 42 °C. The ChIP-DNA was finally purified using the DNA purification kit (A1460, Promega) and used for qPCR. The purified ChIP-DNA was diluted 1:100 and used as a template for the qPCR. Following are the PCR conditions for Alu PCR: 95 °C-5 min, (95 °C- 20 s, 55 °C- 20 s, 72 °C-30 s, 80 °C-30 s) × 50, melting stage. The primers used for Alu PCR were 5′-GAGGCTGAGGCAGGAGAATCG-3′ and 5′-CGCCCAGGC TGGAGTGCAGTGGCGCG-3′. Similarly, CTCF and H3K4me3 ChIP-qPCRs were performed using primers for Alu as indicated above and primers for CTCF-binding sites (5′-CGTAGTTGGGCAGGTTGTCT-3′ and 5′-CAGCTAGGGGGCTACTTCCT-3′) covering the following genomic coordinates in hg38: chromosome 2:128372275-128,372,499. The PCR conditions were the same as those for the Alu except for the annealing temperature of 57 °C.

### Nucleo-cytoplasmic fractionation and Western blotting

The cytoplasmic and nuclear fractions from the HEK293T cells were separated by using the REAP protocol [49]. In brief, HEK293T cells were grown in 10 cm diameter cell culture dishes. Cells were treated with 2 μM Givinostat or DMSO for 24 h. Cells were harvested with a scraper and washed twice with ice-cold PBS. Cell pellets were resuspended in 250 μl ice-cold plasma membrane lysis buffer (PMLB) [PBS with 0.1% NP40 (PI78441, Sigma) and protease inhibitor cocktail (PI78441, Sigma)], mixed 3 times using a p1000 micropipette and centrifuged at 1200 rpm in a benchtop microfuge for 10 s. The supernatant was removed. The supernatant was centrifuged again at 1200 rpm 10 s and the clarified supernatant was used as the cytoplasmic fraction. The pellet was washed with PMLB and lysed in 250 μl ice-cold RIPA buffer (150 mM NaCl, 5 mM EDTA, 50 mM Tris (pH 8.0), 0.1% Sodium deoxycholate, 1% NP-40, 0.5% SDS) containing 1x Halt Protease Inhibitor cocktail (PI78441, Sigma) for 30 min. These nuclear lysates were cleared by centrifugation. Equal volumes of the cytoplasmic and nuclear fractions were subjected to SDS-PAGE followed by Western blotting. The levels of CGGBP1 in nuclear and cytoplasmic fraction were compared between mock (DMSO-treated) or Givinostat-treated HEK293T cells. GAPDH and Histone 3 antibodies (H3K4me3, H3K27me3, H3K9me3 and total Histone H3) were used to identify cytoplasmic and nuclear fractions respectively.

### Histone extraction

HEK293T cells were grown in 10 cm cell culture dishes. Cells were harvested with a scraper and washed twice with ice-cold PBS. Cells were resuspended in Triton Extraction Buffer (TEB: PBS containing 0.5% Triton X 100 (v/v) and protease inhibitor cocktail (PI78441, Sigma)). Cells were lysed on ice for 10 min and centrifuged at 6500 x g for 10 min at 4 °C. The supernatant was discarded and the nuclei in the pellet were washed with TEB and centrifuged at 6500 x g for 1 min at 4 °C. The pellet was resuspended in 250 μl of 0.2 N HCl and left for acid extraction overnight at 4 °C. Nuclear extracts were centrifuged at 6500 x g for 10 min at 4 °C and the clear supernatant was neutralised with 1/10 volume of the 2 M NaOH. The pH of the neutralised extracts was adjusted to 7.5 by the addition of 1 M Tris pH 6.8 and finally diluted 10 times with 1xPBS for DBID assays.

### DNase activity determination

Genomic DNA was isolated from HEK293T cells. Genomic DNA (2 μg) was incubated with Givinostat (100 μM) or DNaseI (2 units) at room temperature for 5, 10 or 15 min. As a control, the same amount of genomic DNA was incubated for 15 min at room temperature without any Givinostat or DNaseI. The treatments (Givinostat or DNaseI) were quenched by adding 1 μl of 0.5 M EDTA to all samples. The degradation activity of the Givinostat was determined by agarose gel electrophoresis.

## Results

### An assay combining **D**ot-**B**lot and **I**mmuno**D**etection (DBID) detects in vitro DNA-protein interactions

To develop a method for finding out a chemical inhibitor of DNA binding and testing it on CGGBP1, we needed to conform to the following preconditions: (i) detecting sequence-independent DNA-binding and its inhibition, (ii) the DNA-binding shall include binding to repeats, and (iii) no requirement of any prior knowledge about the structure of the target protein (in this case, the structure of CGGBP1 is not reported). In addition, we sought to develop a method that would be applicable to any combination of DNA and protein and be based on simple principles of biochemistry that can be inexpensively customized as per experimenters’ choice. We devised a simple assay to screen for inhibitors of protein-DNA interactions by borrowing the principles of Southwestern blotting [50, 51] and immunochemistry [52, 53] and applied it for CGGBP1.

**Fig. 1:**
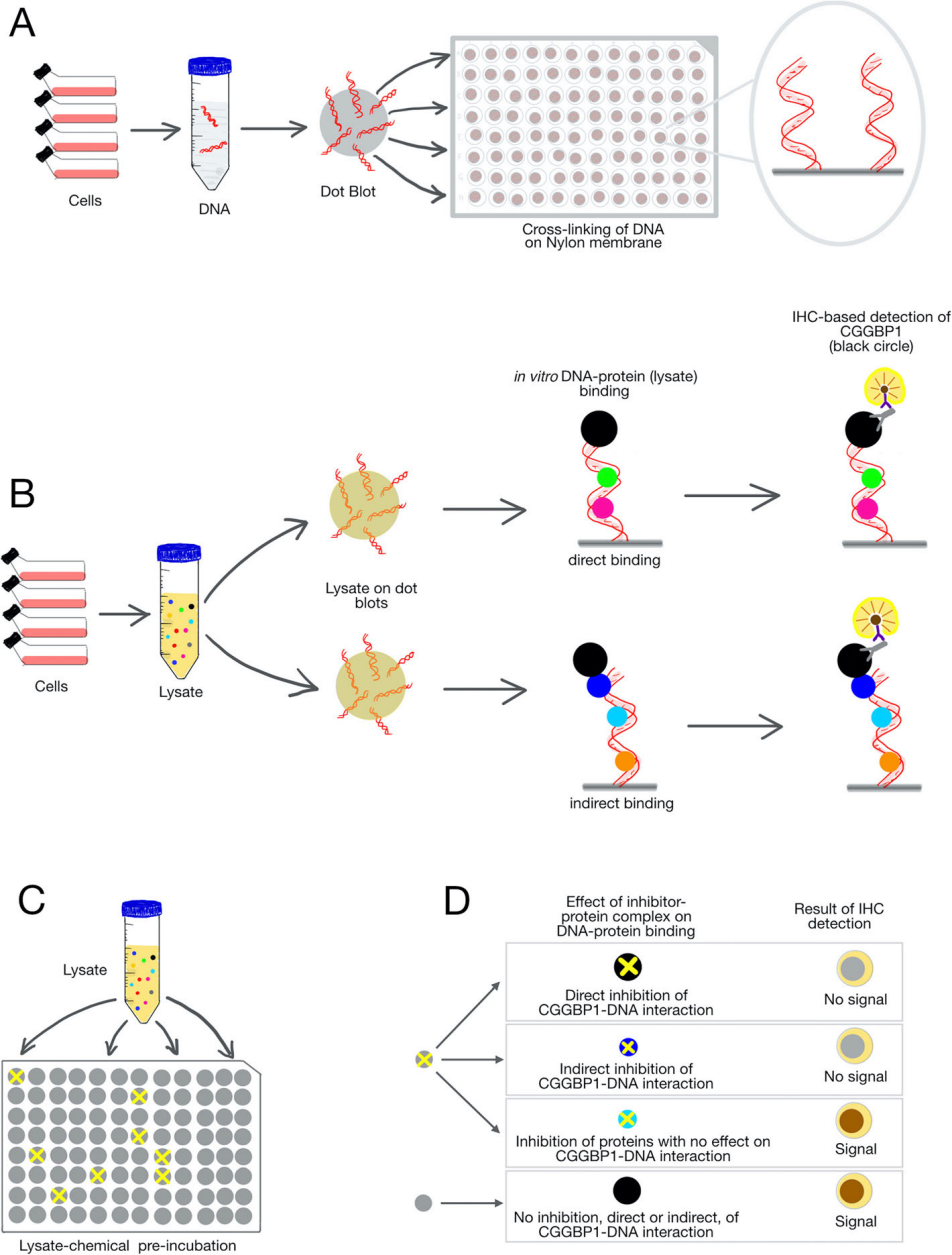
A schematic representation of the **D**ot-**B**lot and **I**mmuno**D**etection (DBID) assay. **a**: Genomic DNA was isolated from HEK293T cells. Sonicated DNA fragments (mean length of 1 kb) were blotted onto positively charged nylon membranes and crosslinked by vacuum heating at 80 °C. **b**: HEK293T cells were lysed and the cleared lysates were used as a source of protein for in vitro DNA binding. The assay depicted here is designed to detect binding of CGGBP1 (black circle) to DNA, although this protocol can be generically applied for any protein of interest. CGGBP1 can bind to DNA either directly (top panel) or indirectly (through linker proteins, depicted in the blue circle in the bottom panel). The subsequent immunochemistry-based detection reports a brown signal for the direct as well as indirect CGGBP1-DNA complexes alike. The immunochemistry employs a primary antibody against the protein of interest, a biotinylated secondary antibody, streptavidin-HRP conjugates and DAB as the chromogenic substrate and is semi-quantitative in nature. **c**: Pre-incubation of the cell lysate with inhibitors allows the small molecule compounds to bind to their cognate target proteins in the lysate. Only some of these compounds potentially inhibit the DNA-binding of their target proteins (exemplified with a yellow cross). **d**: The different possible outcomes of the DBID assay for inhibition of CGGBP1-DNA interactions are depicted. The direct inhibition of CGGBP1 (black circle with a cross) as well as the inhibition of a linker protein (blue circle with a cross) required for CGGBP1-DNA binding are expected to result in “No signal”. Inhibitors that do not have any direct or indirect effect on CGGBP1-DNA interaction show “Signal”

Genomic DNA was extracted from rapidly growing cultures of HEK293T and sonicated to generate ~ 1 kb (mean length) long fragments. Positively charged discs of nylon membranes were soaked in sonicated genomic DNA solution and the DNA was covalently attached to the nylon membrane discs by vacuum baking at 80 °C for 90 min without any denaturation (Fig. 1a). An estimated 0.5 μg of DNA was immobilized on each membrane disc. The DNA-linked membrane discs were blocked using the fetal bovine serum in phosphate-buffered saline supplemented with triton X-100 and subsequently used as probes to capture proteins from cellular lysates. The incubation of DNA-coated membrane discs with concentrated cellular lysates allows capturing of DNA-binding protein complexes present in the lysates (Fig. 1b). These DNA-coated and crosslinked membrane discs are henceforth referred to as “dot blots”.

Lysates were prepared from HEK293T cells as described in the methods. Proteins in the lysates were pre-incubated with the compounds in the library at a uniform concentration of 100 μM (or 0 μM as a negative control for inhibition) (Fig. 1c). Subsequently, the lysate-inhibitor mixtures were incubated with the dot blots in a binding buffer for 90 min at 4 °C. The dot blots were washed in PBS and crosslinked using 4% formaldehyde. The fixed DNA-protein complexes on the dot blots were probed with a primary anti-CGGBP1 antibody followed by immunohistochemical detection (Fig. 1d). For library-scale detection, the dot blots were serially incubated with biotinylated secondary antibody, streptavidin-horseradish peroxidase (HRP) conjugate and chromogenic HRP substrate that included hydrogen peroxide. The signals were visually evaluated to select strong inhibitors. For further screening of a panel of primary hits from the library screen, HRP-conjugated secondary antibodies were used directly followed by chemiluminescent detection. The signals on the secondary dot blots were quantified using densitometry of inverted images of the blots.

First, we ensured that the detection was specific by using negative controls. The chromogenic signal detected on the dot blots using the anti-CGGBP1 antibody was specific as the signal was reduced to a weak background when the primary antibody was replaced with isotype-control IgG or serum. Similarly, replacing the lysate with the blocking buffer diminished the signal.

### Library screen for identification of CGGBP1 inhibitors using DBID

**Fig. 2:**
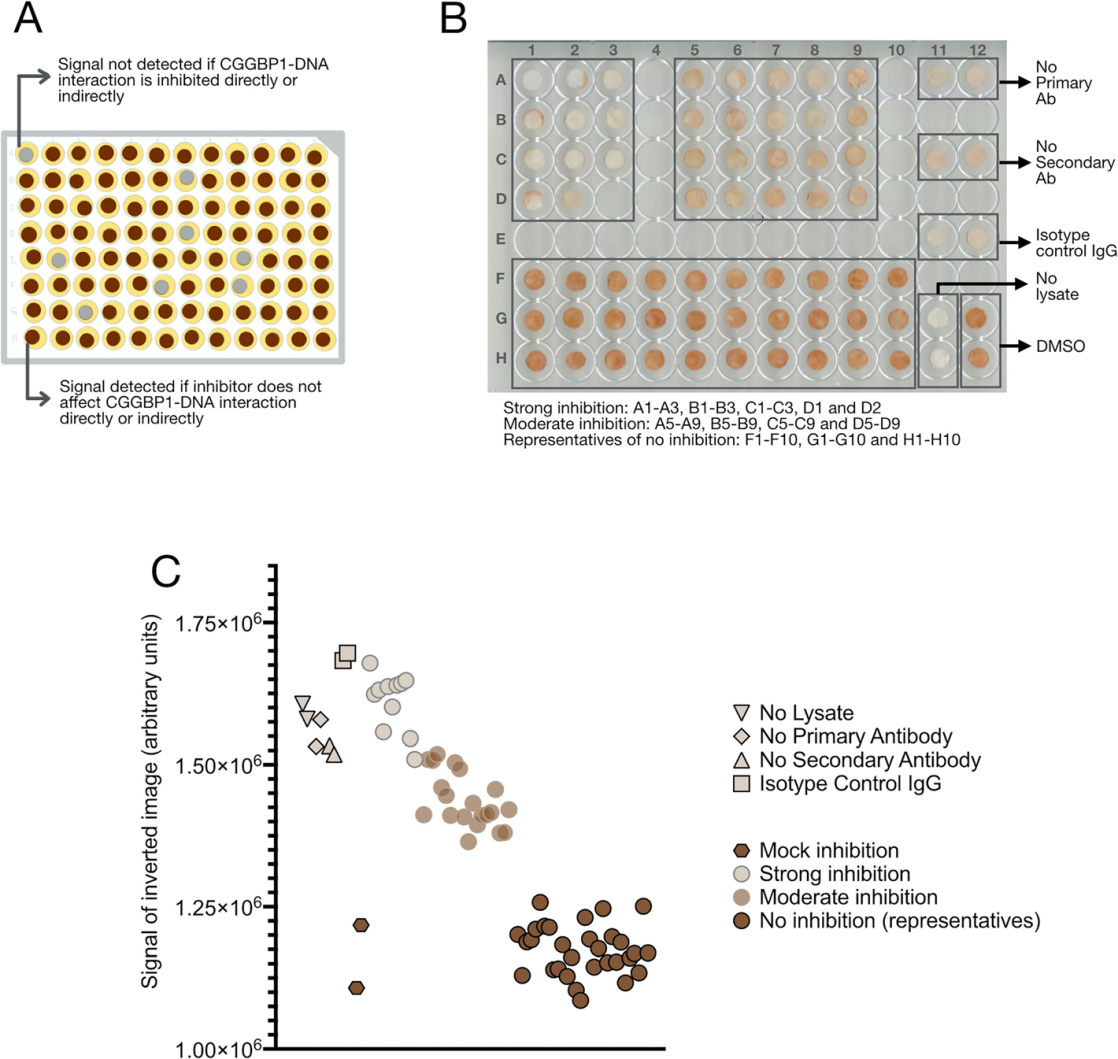
DBID screening of a small molecule chemical library (1685 compounds) identifies inhibitors of CGGBP1-DNA interaction. **a**: The primary DBID assay was performed in multiple 96-well plates. The lysate was individually pre-incubated with the compounds (one compound per well). After transferring the lysate-compound complexes (as shown in Fig. 1c) to dot blots (as shown in Fig. 1a), immunochemical detection was performed using a cocktail of rabbit anti-CGGBP1 primary antibody. The schematic represents the signals obtained for a 96-well plate. **b**: The dot blots of the entire library screen for CGGBP1 were manually categorized into 11 strong inhibitors and 20 moderate inhibitors. The actual images of these two groups of dot blots are shown here along with the positive and negative controls as indicated. The identities of the inhibitors are as follows: Strong inhibitors [A1-Givinostat (ITF2357), A2-LRRK2-IN-1, A3-Peficitinib (ASP015K, JNJ-54781532), B1-Ispinesib (SB-715992), B2-TWS119, B3-Domperidone, C1-Gallamine Triethiodide, C2-Moxifloxacin HCl, C3-Sirtinol D1-NAD+, D2-Palbociclib (PD-0332991) HCl], Moderate inhibitors [A5-VX-661, A6-BRD73954, A7-NCT-501, A8-Tenovin-1, A9-Prasugrel, B5-U0126-EtOH, B6-Foretinib (GSK1363089), B7-JNJ-7706621, B8-CHIR-99021 (CT99021), B9-Asenapine maleate, C5-Ethylparaben, C6-LY2874455, C7-Golgicide A, C8-PD173955, C9-Mirin, D5-Ramelteon, D6-Cilnidipine, D7-Dopamine HCl, D8-VR23, D9-AZD3759]. Majority of the compounds in the library did not show any inhibition of CGGBP1-DNA interaction. Thirty representative dot blots of the non-inhibitors are shown in the well numbers F1-F10, G1-G10, H1-H10. **c**: The signals for the dot blots shown in B are quantified by densitometry. The graph shows the signals of the inverted images for each dot blot

To identify inhibitors of CGGBP1-DNA interaction, we pre-incubated the HEK293T cell lysates with inhibitors from a library of bioactive compounds before allowing the capture of protein complexes by DNA-bound dot blots. Pre-incubation with any compound that successfully inhibited DNA-CGGBP1 interactions would result in a weaker signal using an anti-CGGBP1 antibody as compared to a mock inhibition (only the compound dilution buffer) (Fig. 2a). The lysate was incubated with each compound separately at a final concentration of 100 μM. The parallel nature of compound-protein incubation in this protocol made it convenient to use one single concentration for all the compounds in the library. As DNA-binding inhibition requires higher drug concentrations [22], we settled on a concentration of 100 μM for the screen library-wide. Since this concentration was only for the in vitro screening, wherein the hits would be subjected to verification experiments, we chose this concentration. Thus, the choice of 100 μM chemical concentration was likely to eliminate false negatives whereas the subsequent verification experiments for the screen hits at lower concentrations would allow us to eliminate false positives. Majority of the compounds in the library showed no inhibition of CGGBP1-DNA binding, whereas a subset of compounds displayed moderate to strong inhibition of CGGBP1-DNA binding (Fig. 2b and c). The 1685 dot blots, one for each compound of the library, were categorized into three groups: no inhibition for 1554 compounds (strong and consistent signals comparable to mock inhibition), moderate inhibition for 20 compounds (relatively weaker signal compared to mock inhibition), and strong inhibition for 11 compounds (negligible to very weak signals compared to mock inhibition) (Fig. 2b). For 100 compounds the assay was inconclusive and we eliminated them from the analysis.

Although we could identify some inhibitors of CGGBP1-DNA interaction, it remained unclear if the inhibition were direct or indirect. We pursued the inhibitors identified in this primary screen further to detect direct inhibitors of CGGBP1-DNA interactions.

### Application of DBID to identify direct inhibitors of CGGBP1

**Fig. 3:**
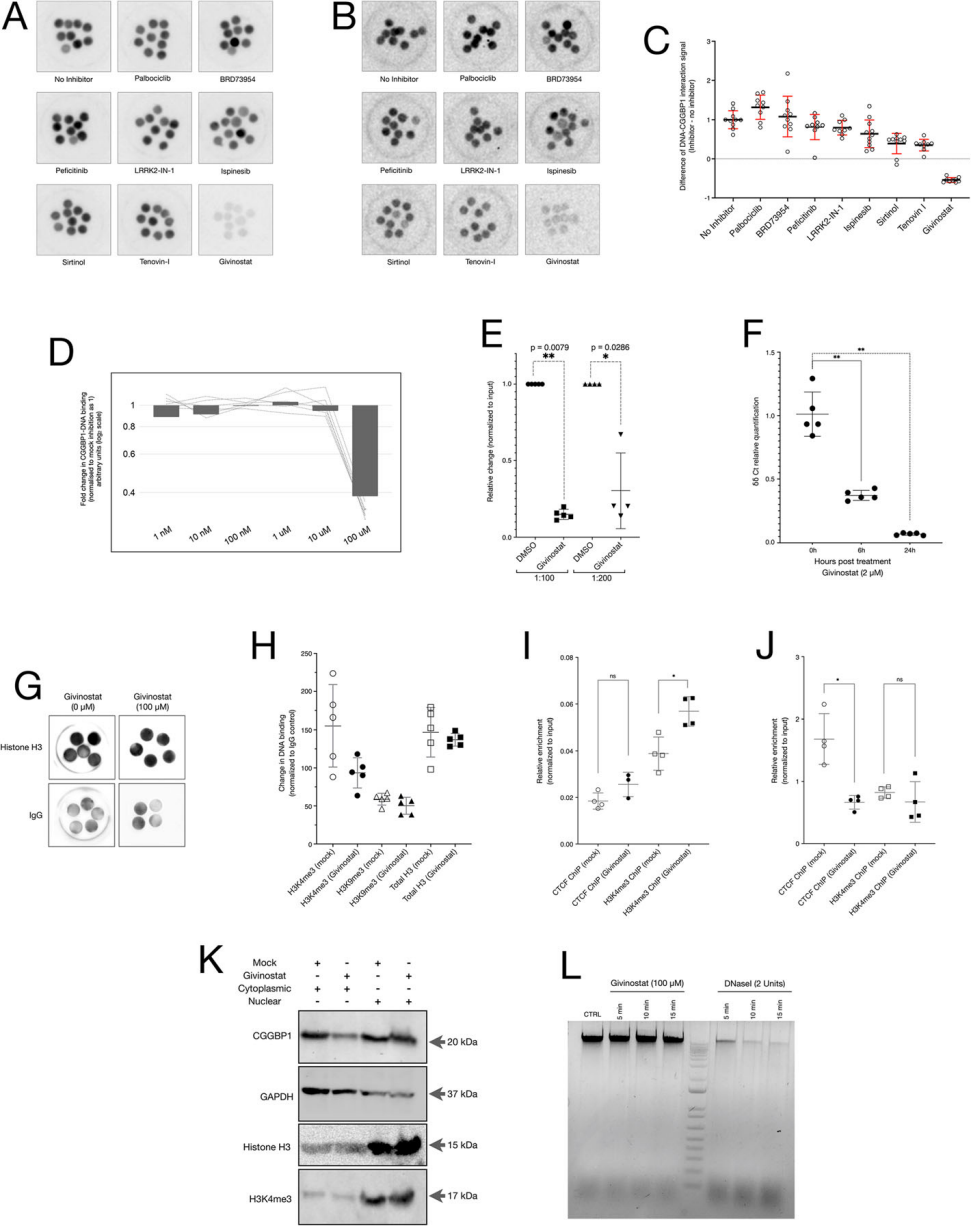
Givinostat acts as a specific direct inhibitor of rCGGBP1-DNA interaction in vitro. A and B: DBID using anti-FLAG (**a**) or anti-CGGBP1 antibodies (**b**) against rCGGBP1 pre-incubated with compounds as indicated show a direct inhibition by Givinostat. The multiple dot blots per compound are technical replicates. **c**: Quantification of the signals obtained from the dot blots of secondary screening (**b**) are plotted. D: Densitometric signals of DBID assay for CGGBP1 using lower concentrations of Givinostat show no inhibition at concentrations lower than 100 μM. **e**: Pre-incubation of rCGGBP1 with Givinostat reduces the amount of Alu DNA co-precipitating with FLAG-antibody by 70-80%. **f**: An input-normalized δδCt relative quantification of CGGBP1 ChIP DNA from HEK293T cells treated with 2 μM Givinostat or DMSO for the indicated durations shows inhibition of CGGBP1 occupancy at Alu elements by Givinostat. **g**: The DBID assay for acid-extracted nuclear proteins using total histone H3 antibody shows no inhibition of DNA-binding of histone H3 by Givinostat. **h**: DBID signals (background-subtracted and normalized for IgG signals) for the H3K4me3, H3K9me3 and total histone H3 show no significant change due to 100 μM Givinostat. **i** and **j**: qPCRs on CTCF and H3K4me3 ChIP-DNA show that H3K4me3 is significantly increased at Alu elements (**i**) whereas CTCF occupancy at a CGGBP1-regulated CTCF-binding site is significantly decreased (**j**) by Givinostat treatment. * indicates Mann-Whitney *p*-value < 0.05; ns represents *p*-value > 0.05. **k**: Nuclear-cytoplasmic fraction analysis of mock- or Givinostat-treated HEK293T cells showed that the nuclear levels of CGGBP1 and histone H3 remain unchanged upon Givinostat treatment. GAPDH serves as a loading control for the cytoplasmic fraction. **l**: A comparison of Givinostat or DNaseI treated genomic DNA shows Givinostat does not cause the degradation of DNA while DNaseI digests DNA into low molecular weight fragments and increases the low molecular weight smear

To identify the direct inhibitors of CGGBP1-DNA binding, the next set of experiments were performed on recombinant CGGBP1 (rCGGBP1) containing a C-terminal FLAG tag and a panel of available inhibitors identified in the primary screen. The dot blots were blocked with blocking buffer. Similarly, instead of the lysates, rCGGBP1 was incubated for 30 min with the inhibitors (100 μM) identified from the primary screen. The rCGGBP1-inhibitor mix was incubated with the blocked dot blots and probed with anti-FLAG or anti-CGGBP1 antibodies followed by HRP-conjugated secondary antibody incubation and chemiluminescent signal detection (Fig. 3a and b) (raw data in Additional files 1, 2, 3, 4, 5 and 6). Signals were quantified and analysed through densitometry (Fig. 3c). The inhibitors used in this secondary DBID assay could be again classified as having (i) low or no detectable direct inhibition (Palbociclib, BRD73954, Peficitinb, LRRK2-IN-1 and Ispinesib) or (ii) moderate inhibition (Sirtinol and Tenovin-I). Only one compound, Givinostat, displayed a strong inhibition of rCGGBP1-DNA interaction (Fig. 3c).

To establish the effect of lower concentrations of Givnostat on CGGBP1-DNA interaction, the assay was re-performed on a series of Givnostat concentrations (10 μM, 1 μM, 0.1 μM, 0.01 μM and 0.001 μM). Concentrations lower than 100 μM did not yield detectable inhibition in DBID (Fig. 3d) (raw data in Additional file 7). However, this could partly be due to a low dynamic range in the DBID signals.

### Verification of CGGBP1-DNA interaction inhibition by Givinostat

We next verified the inhibition of rCGGBP1-DNA interaction using an alternative approach. Alu DNA is a strong binding site for CGGBP1 and interactions between Alu DNA and CGGBP1 have been demonstrated earlier in vitro as well as in vivo [32].

We performed in vitro immunoprecipitation of Alu DNA using rCGGBP1 with or without inhibition by Givinostat. The difference in the amount of Alu DNA immunoprecipitated using the mock-inhibited rCGGBP1 or Givinostat-inhibited CGGBP1 was quantified using real-time PCR (Fig. 3e). Using the amount of input Alu DNA as a control, we could establish that Givinostat is a strong direct inhibitor of CGGBP1-Alu DNA interaction.

Further, the inhibition of CGGBP1-DNA interaction by Givinostat was validated by the ChIP-qPCR for binding sites of CGGBP1. ChIP was performed for CGGBP1 on HEK293T cells treated with 2 μM Givinostat for different time-points (6 h and 24 h). Givinostat caused inhibition of CGGBP1-DNA binding as evident from the ChIP-qPCR amplification for Alu at 6 h compared to 0 h (mock inhibited, DMSO). This inhibition was significantly increased upon treatment of cells with Givinostat for 24 h (Fig. 3f).

To rigorously test the limits of the DBID assay, we needed to test the protocol on DNA-binding of a protein presumptively not inhibited by Givinostat. We chose to apply DBID on histone H3 and its post-translationally modified forms H3K9me3 and H3K4me3. Although histones H3 and CGGBP1 are unrelated proteins, they both have comparable molecular weights and bind to DNA motif-independently. Moreover, H3K9me3 and H3K4me3 are differently regulated by CGGBP1 depletion in HEK293T cells [41]. Thus, we compared the findings with ChIP-qPCR assays on H3K4me3 and CTCF, a known target protein of CGGBP1 [41].

DBID assay using a total histone H3 antibody against acid extracted proteins from purified nuclei showed that Givinostat did not inhibit the DNA-binding of histone H3 (Fig. 3g) (raw data in Additional files 8 and 9). A similar analysis with H3K4me3 and H3K9me3 and its comparison with total histone H3 showed that Givinostat did not inhibit the DNA binding of these forms of histone H3 (Fig. 3h) (raw data in Additional file 10). Although some technical replicates in H3K4me3 DBID assay showed a wide variation of DBID signal, none of these was significant (Mann-Whitney test; *p* > 0.05). ChIP for CTCF or H3K4me3 followed by qPCRs were employed to test if in HEK293T cells a CGGBP1 inhibition by Givinostat could mimic the already known effects of CGGBP1 depletion by shRNA. Givinostat treatment did not affect CTCF occupancy but significantly increased H3K4me3 levels at Alu elements (Fig. 3i). Conversely, at a newly determined CGGBP1-dependent CTCF binding site at which H3K4me3 is not affected by CGGBP1 depletion, we could establish that Givinostat treatment reduced CTCF occupancy without any significant change in H3K4me3 levels (Fig. 3j).

Western-blot analyses of nuclear and cytoplasmic fractions of mock- or Givinostat-treated HEK293T cells showed that the levels of CGGBP1 in the nuclear fraction were not reduced due to Givinostat treatment (Fig. 3k) (raw data in Additional files 11 and 12). Similarly, the levels of total histone H3 or H3K4me3 in the nuclear fraction were not affected by Givinostat treatment (Fig. 3k). Thus, the reduction in CGGBP1 occupancy at Alu elements, CTCF occupancy at CGGBP1-regulated CTCF-binding sites and an increase in H3K4me3 at Alu elements was not due to changes in subcellular distributions of these proteins. Additionally, we also ensured that the Givinostat-induced inhibition of DNA binding observed in the DBID assays and the in vitro DNA IPs were not due to degradation of DNA. High molecular weight genomic DNA from HEK293T cells was incubated with Givinostat (to mimic the DBID incubation conditions) or DNaseI (positive control for DNA digestion). While DNaseI digested the DNA and generated low molecular weight smear, no degradation of DNA was observed by incubation with Givinostat (Fig. 3l) (raw data in Additional file 13).

## Discussion

The DBID method presented here offers several advantages of cost, simplification and generic applicability over other methods reported in the past [22, 24]. The first advantage of the method is that it does not require special equipment or reagents. The dot blots could be generated using genomic DNA, PCR products, linearized plasmids or oligonucleotides. Using genomic DNA would allow an unbiased assessment of binding and its inhibition, especially relevant for proteins about which DNA-binding site information does not exist. The length of the DNA fragments used on the discs is also easily changeable without any challenges. Longer genomic DNA fragments offer the advantage of the formation of macromolecular structures that are important regulators of DNA-binding. We have used ~ 1 kb long genomic DNA fragments in this assay and this length of DNA is sufficient to allow the formation of complex structures of DNA that would not be possible if the method were restrictive for DNA length and sequence. Labelling of DNA, such as biotinylation, is an expensive component, especially if needed at a library screening scale. The usage of unlabelled DNA makes the DBID assay suitable for very large libraries as well as unlabelled DNA is cheaper and easier to generate.

Conventionally, the small molecule inhibitors of enzyme activities (where the substrate to product conversion a standard readout of enzyme function) are easier to test because the enzyme assays can provide a direct measurement of the inhibition. Assuming that CGGBP1 is a DNA-binding protein with no enzymatic activity, a similar measurement of CGGBP1 “activity” and the “inhibition of its activity by a compound” is not possible. Thus, we had to first define the “activity” of CGGBP1 and then invent a methodology to screen for inhibitors. Building upon the knowledge that CGGBP1 is a DNA-binding protein, we worked under the constraints of an assumption that DNA-binding of CGGBP1 is required for its functions. As a corollary, an inhibition of DNA-binding of CGGBP1 could be stated as inhibition of CGGBP1. However, CGGBP1 is a protein with myriad functions and yet its structure remains uncharacterized. Although predicted structures of CGGBP1 using homology modelling software allow insights into its possible structures, this information is unreliable for rational inhibitor design. Interestingly, CGGBP1 is highly thermolabile, adding another level of uncertainty to any assumptions about its structure-activity relationship. In light of the above-mentioned challenges, we established the DBID protocol as a generic method to screen a library of inhibitors. The advantages of such a protocol are that it is simpler than previously described methods [21, 22, 24] and can be used for a library level inhibitor screen against any DNA-binding protein, such as transcription factors, for which reliable antibodies are available.

We have performed the DBID assay for human CGGBP1 and the binding conditions were optimized for CGGBP1-DNA binding. The application of DBID to other proteins will require some standardization of the binding conditions. These challenges exist in other in vitro DNA-protein interaction assays, such as electrophoretic mobility shift assays. The DBID assay sensitivity and specificity depends on the antibody used against the target protein. The choice of antibodies and immunochemistry is also flexible in DBID assay. For the library scale screening where imaging and detection using chemiluminescent substrate is not preferred, we could use a combination of primary antibody-biotinylated secondary antibody-streptavidin HRP conjugates and DAB-hydrogen peroxide-based chromogenic signal generation. This allowed a quick visual assessment of the signals. In our experience, the chromogenic signal remains stable on the dry membranes and can be revived after several weeks by moistening of the membranes. The signal amplification steps minimize the detection of weak inhibition of DNA-protein interactions. At the same time, only the very strong inhibition would fail to generate DBID signals. Thus, the assay is poor in sensitivity for detection of weak inhibitors but robust as the detection method is biased against false positives.

While using the rCGGBP1 as the target protein for a direct inhibition detection by selected primary hits, we could apply a combination of primary antibody-secondary antibody conjugated with HRP followed by enhanced chemiluminescent substrate-based detection. These are common reagents widely used in Western blotting and add to the versatility of the DBID assay. We could successfully use DBID for CGGBP1 in whole-cell lysates, pure rCGGBP1 and acid-extracted histone H3 suggesting that the combination of binding buffer and incubation conditions as well as the detection protocol would likely work for a range of target proteins and protein purification methods. We also used DBID for H3K9me3 and H3K4me3. Using the antibodies against H3K9me3 and H3K4me3, which worked reliably in Western blotting, we had to deal with some uneven background in the DBID assay. Since the chemiluminescence signals are quantifiable, we could perform background subtraction on these blots followed by normalization using signals derived from an IgG control. The DBID findings could be verified by qPCR on in vitro DNA-IPs and ChIP-DNA. The reproducibility of the findings using ChIP is particularly important as we could specifically test the effects of CGGBP1 depletion on its own binding to target DNA (Alu) as well as on CGGBP1-regulated CTCF-binding sites. Put together, the DBID assay offers the advantages of a large scale screen for identifying DNA-binding inhibitors with the simplicity of routine Western blotting and immunochemistry. The assay is scalable, adaptable by changing the target protein-antibody combination and can be used to test different types of DNA rapidly.

The application of DBID to any target protein will need some optimization owing to the nature of the protein, knowledge about the DNA sequences it binds to and the antibodies used. An evaluation of DBID in the context of the DNA-binding properties of CGGBP1 helps us better identify its merits and limitations. CGGBP1 binding to DNA has been shown to be sensitive to cytosine methylation at CGG repeats. However, it remains unknown if the cytosine methylation of Alu DNA, that we have used for direct rCGGBP1-DNA interaction inhibition analysis, also serves as an inhibitor of Alu DNA binding of CGGBP1. We generated methylated or unmethylated Alu DNA by using dNTP mixes containing either methyl-dCTP or unmethylated dCTP. Interestingly, the binding of rCGGBP1 to the Alu DNA did not change even if all cytosines were methylated (not shown). These results indicate that the inhibition of direct CGGBP1 binding to Alu DNA by Givinostat is not affected by the cytosine methylation status of the Alu DNA. The control DBID and ChIP-qPCR assays with histones, specifically H3K4me3, clearly show that the DBID assay helps us differentiate between the direct and indirect effects of Givinostat treatment. The non-inhibition of histone-DNA binding by Givinostat in vitro combined with specific inhibition of downstream targets of CGGBP1 (CTCF at CGGBP1-regulated binding sites and H3K4me3 at Alu elements) in cells establishes the validity of our findings.

DNA-binding proteins regulate a vast array of cellular functions. Transcription, replication, DNA repair, chromatin structure and function are governed by DNA-protein interactions, many of which are not DNA sequence motif-dependent. These processes are frequently deregulated in cancer and application of the DBID assay to find out chemical inhibitors of DNA-binding proteins opens up hitherto unexplored possibilities in cancer research. Our findings of inhibition of CGGBP1-DNA interaction by Givinostat also suggest that DBID assay will reveal molecular repurposing of some chemical inhibitors.

## Conclusions

The DBID assay is a rapid and reliable method to screen chemical libraries for DNA-binding inhibitors. The various parameters of the assay, including the drug concentration used for the screen and the target protein, are easily customizable. The assay applied here for human CGGBP1 and genomic DNA interaction or for rCGGBP1 and Alu DNA has generated findings that could be verified by other targeted methods over a wide range of Givinostat concentrations (100 μM in vitro to 2 μM *in cellulo*). These results establish that DBID assay can be used reliably for identifying inhibitors of DNA-protein interactions. A combination of the primary and secondary DBID assays help identify the inhibitors for indirect or direct DNA-binding from direct DNA-binding. The assay has wide applicability in searching for inhibitors of transcription factors, with no dependence on the protein structure. The repurposing of chemical inhibitors currently in use for modulating DNA-binding activity of proteins will have interesting implications in cancer research.

## Supplementary information

**Additional file 1.** Chemiluminescence scan of DBID blots shown in Fig. 3a. The well marked with a red X symbol contains a sample irrelevant to the experiments described. The names of the inhibitors are indicated at the top of each well.

**Additional file 2.** Chemiluminescence scan at a weaker intensity of DBID blots shown in Fig. 3a. The well marked with a red X symbol contains a sample irrelevant to the experiments described. The names of the inhibitors are indicated at the top of each well.

**Additional file 3.** A white light image of DBID blots shown in Fig. 3a.The well marked with a red X symbol contains a sample irrelevant to the experiments described. The names of the inhibitors are indicated at the top of each well.

**Additional file 4.** Chemiluminescence scan of DBID blots shown in Fig. 3b. The well marked with a red X symbol contains a sample irrelevant to the experiments described. The names of the inhibitors are indicated at the top of each well.

**Additional file 5.** Chemiluminescence scan at a weaker intensity of DBID blots shown in Fig. 3b. The well marked with a red X symbol contains a sample irrelevant to the experiments described. The names of the inhibitors are indicated at the top of each well.

**Additional file 6.** A white light image of DBID blots shown in Fig. 3b. The well marked with a red X symbol contains a sample irrelevant to the experiments described. The names of the inhibitors are indicated at the top of each well.

**Additional file 7.** Chemiluminescence scan of DBID blots used for quantification and plotting in Fig. 3d. The Givinostat concentrations are indicated at the top of each well.

**Additional file 8.** Chemiluminescence scans of DBID blots shown in Fig. 3g. The well marked with a red X symbol contains a sample irrelevant to the experiments described. The antibodies used are indicated.

**Additional file 9.** Chemiluminescence scans of DBID blots shown in Fig. 3g. The antibodies used are indicated.

**Additional file 10.** Chemiluminescence scans of DBID blots used for quantification and plotting in Fig. 3h. The Givinostat concentrations and antibodies are indicated along the columns and rows respectively.

**Additional file 11.** Raw data for Fig. 3k. A white light scan of the nuclear and cytoplasmic fractionation blot shows the molecular weight marker to the left. A dotted line between 26 kDa and 34 kDa bands indicates the line of incision at which the membrane was divided into two parts. As indicated, the top and the bottom parts were separately probed for GAPDH and CGGBP1 respectively. As indicated, the chemiluminescence scans of CGGBP1 and GAPDH blots are shown either alone or as an overlay with the white light image that shows the molecular weight markers.

**Additional file 12.** Raw data for Fig. 3k. As indicated, the chemiluminescence scans of H3K4me3 and total histone H3 blots are shown either alone or as an overlay with the white light image that shows the molecular weight markers.

**Additional file 13.** Raw data for Fig. 3l. Uncropped view of the ethidium bromide-stained agarose gel scan (including the wells and molecular weight marker as indicated) with samples as indicated at the top. The two lanes to the extreme right are empty.

## Data Availability

The datasets used and/or analysed during the current study are available from the corresponding author on reasonable request.

## Abbreviations

DBID: Dot Blot and ImmunoDetection
rCGGBP1: recombinant CGGBP1
