# Supplementary figures and images for: An immunochemistry-based screen for chemical inhibitors of DNA-protein interactions and its application to human CGGBP1
Source: BMC Cancer. 2020 Oct 20;20:1016. doi: 10.1186/s12885-020-07526-5 (PMC7576722; doi:10.1186/s12885-020-07526-5)

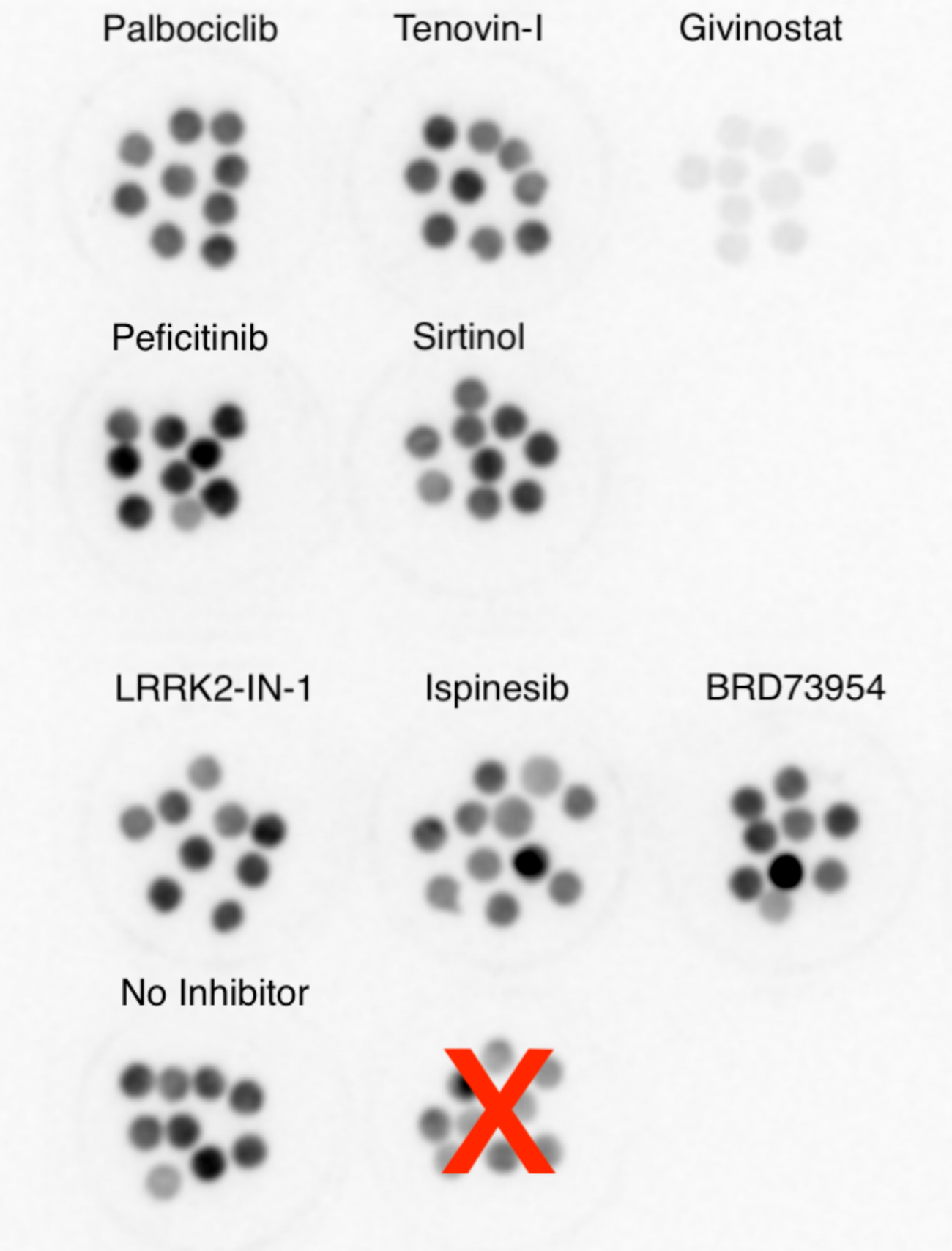

Additional file 1 corresponding to Fig 3A (chemiluminescence, high exposure)

Supplement: Supplementary file 1 — Additional file 1. Chemiluminescence scan of DBID blots shown in Fig. 3a. The well marked with a red X symbol contains a sample irrelevant to the experiments described. The names of the inhibitors are indicated at the top of each well. [file 12885_2020_7526_MOESM1_ESM.pdf]

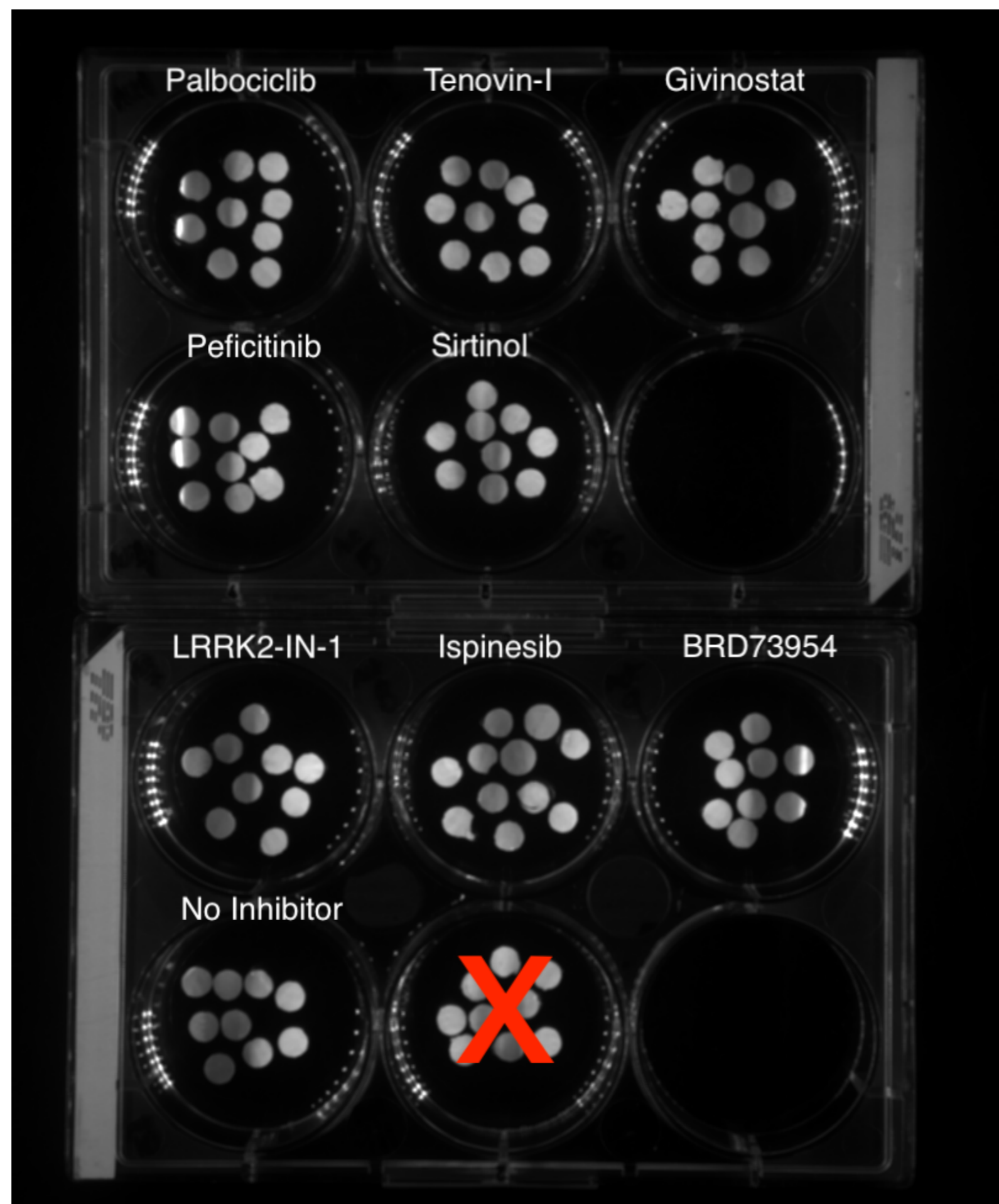

Additional file 3 corresponding to Fig 3A (white light)

Supplement: Supplementary file 3 — Additional file 3. A white light image of DBID blots shown in Fig. 3a.The well marked with a red X symbol contains a sample irrelevant to the experiments described. The names of the inhibitors are indicated at the top of each well. [file 12885_2020_7526_MOESM3_ESM.pdf]

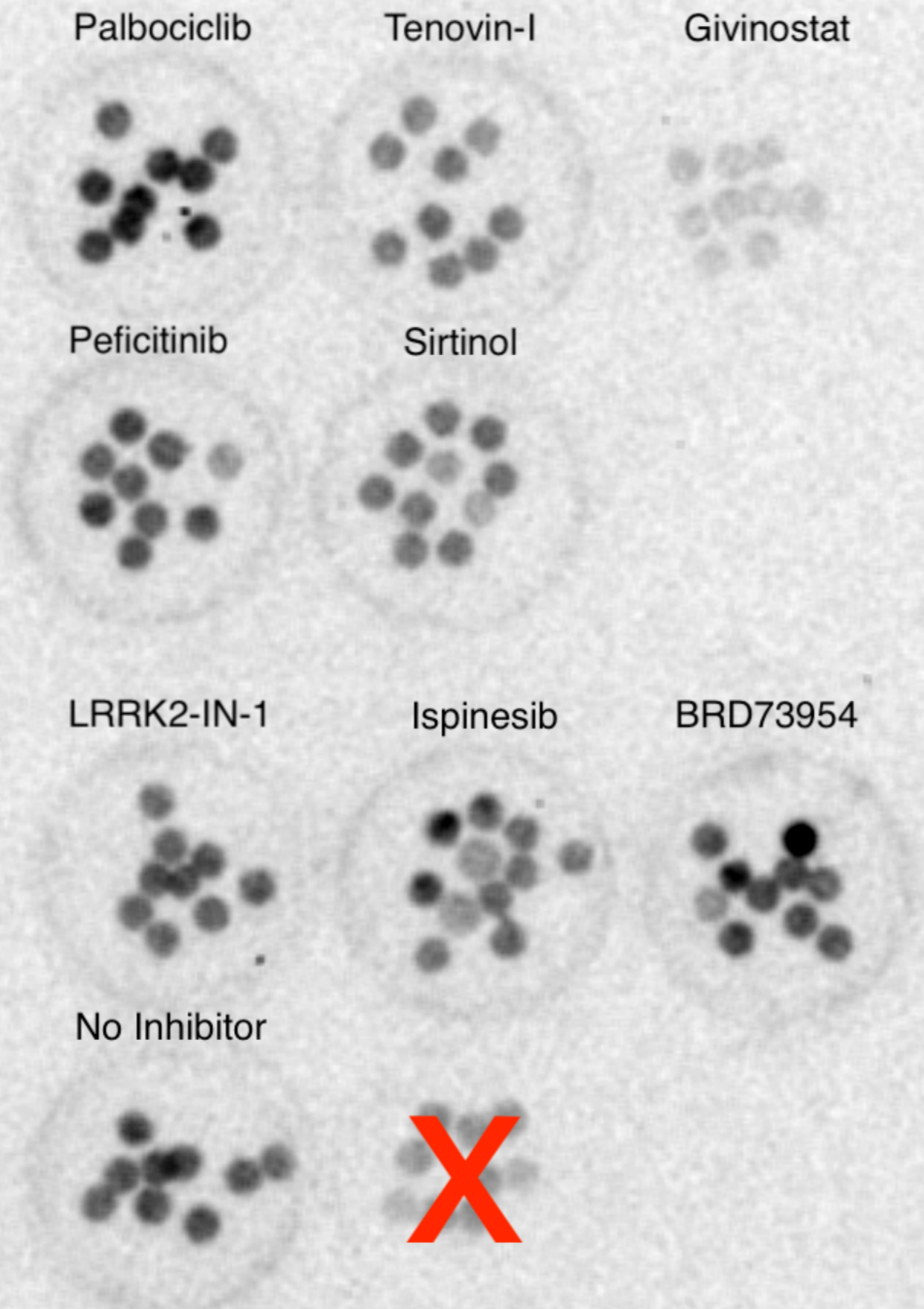

Additional file 4 corresponding to Fig 3B (chemiluminescence, high exposure)

Supplement: Supplementary file 4 — Additional file 4. Chemiluminescence scan of DBID blots shown in Fig. 3b. The well marked with a red X symbol contains a sample irrelevant to the experiments described. The names of the inhibitors are indicated at the top of each well. [file 12885_2020_7526_MOESM4_ESM.pdf]

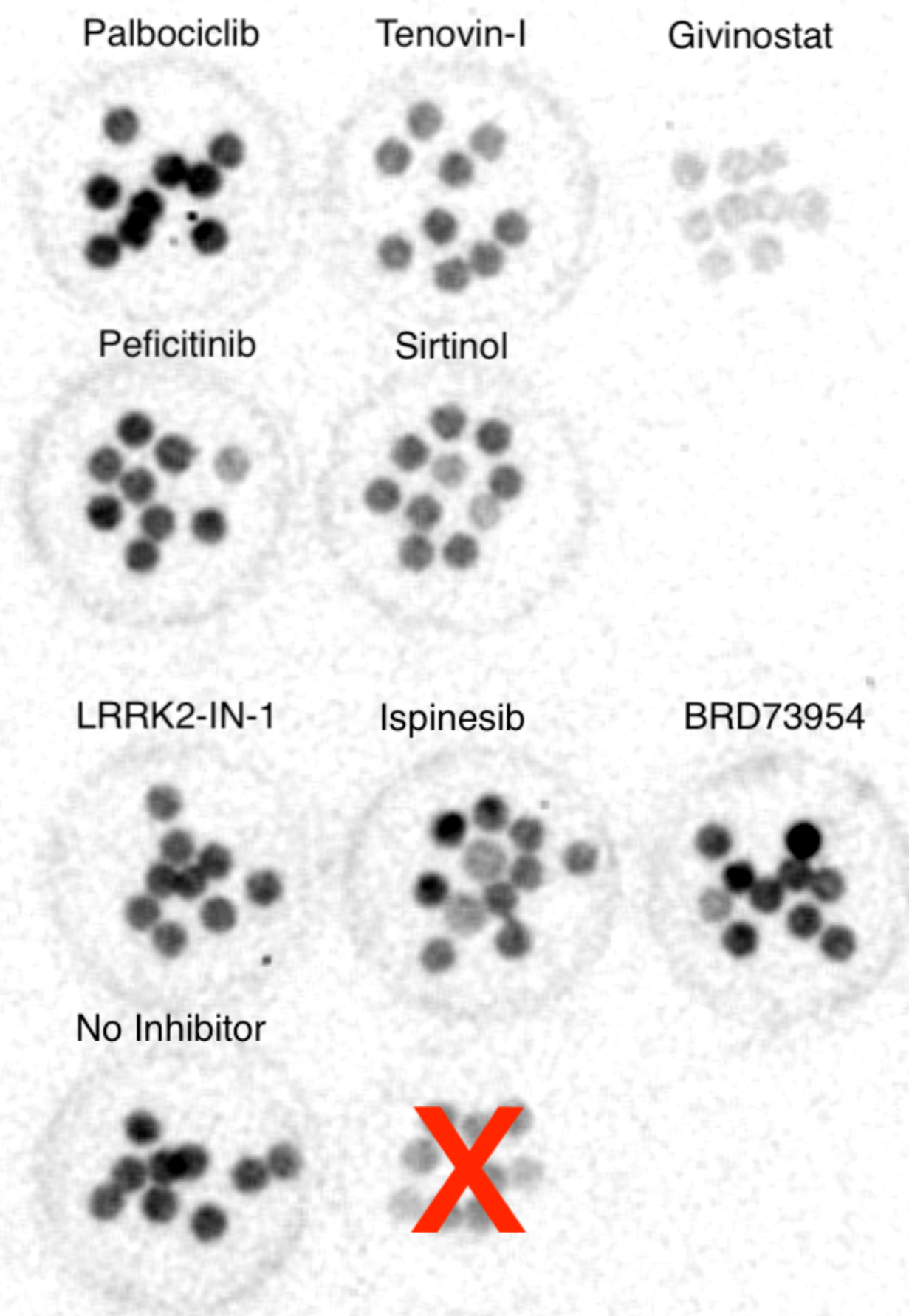

Additional file 5 corresponding to Fig 3B (chemiluminescence, low exposure)

Supplement: Supplementary file 5 — Additional file 5. Chemiluminescence scan at a weaker intensity of DBID blots shown in Fig. 3b. The well marked with a red X symbol contains a sample irrelevant to the experiments described. The names of the inhibitors are indicated at the top of each well. [file 12885_2020_7526_MOESM5_ESM.pdf]

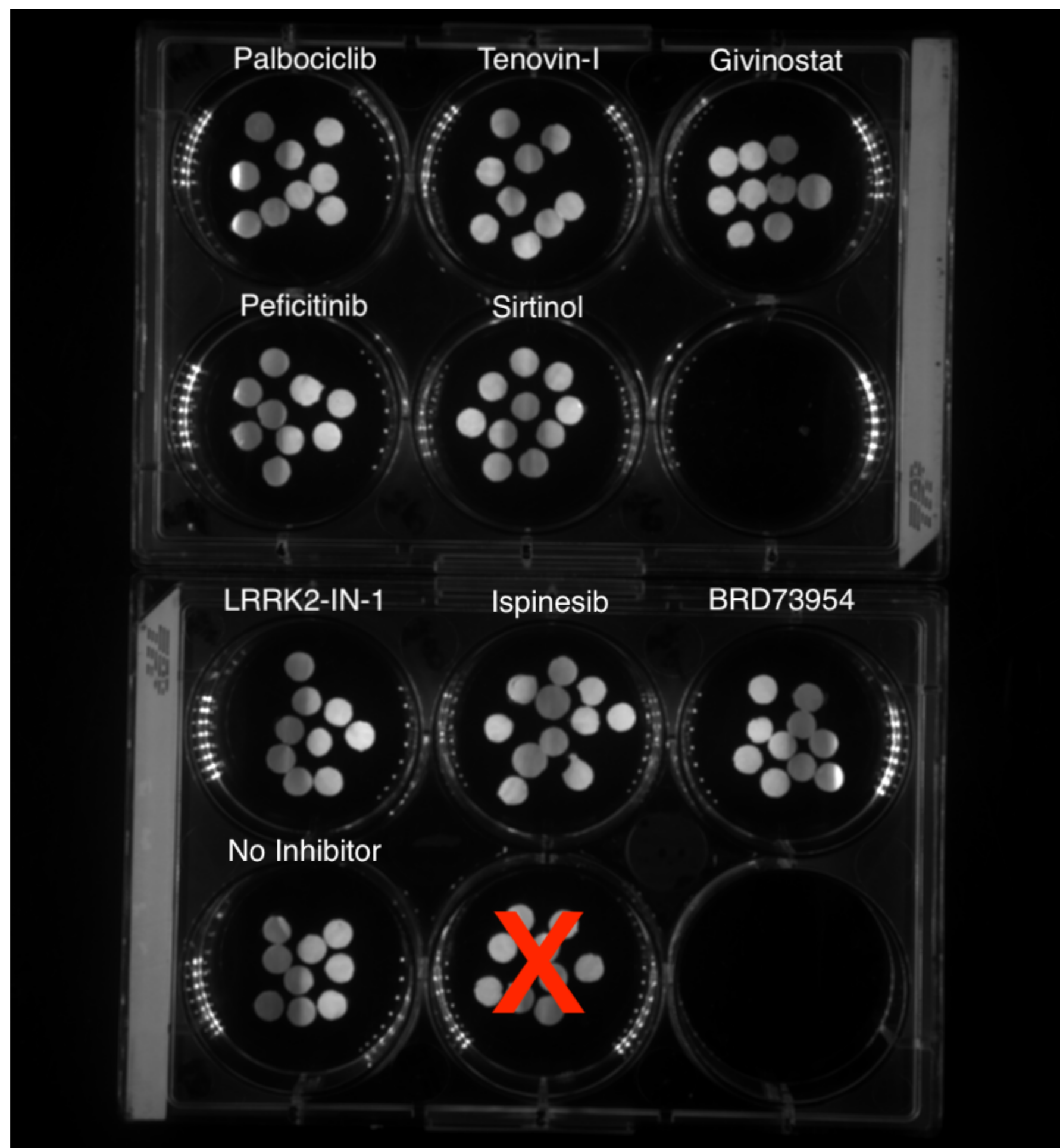

Additional file 6 corresponding to Fig 3B (white light)

Supplement: Supplementary file 6 — Additional file 6. A white light image of DBID blots shown in Fig. 3b. The well marked with a red X symbol contains a sample irrelevant to the experiments described. The names of the inhibitors are indicated at the top of each well. [file 12885_2020_7526_MOESM6_ESM.pdf]

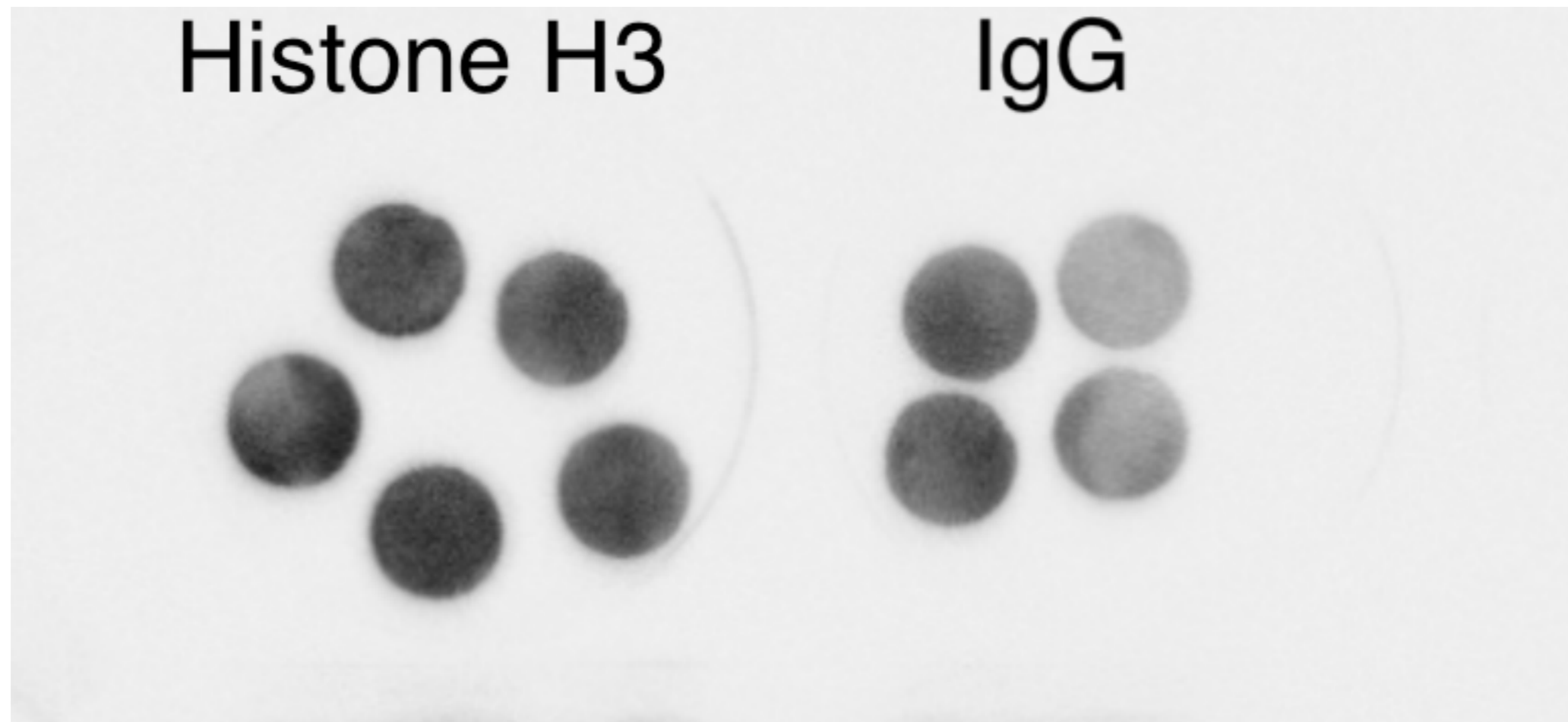

Additional file 9 corresponding to Fig 3G and quantification in Fig 3H  
(chemiluminescence)

Supplement: Supplementary file 9 — Additional file 9. Chemiluminescence scans of DBID blots shown in Fig. 3g. The antibodies used are indicated. [file 12885_2020_7526_MOESM9_ESM.pdf]

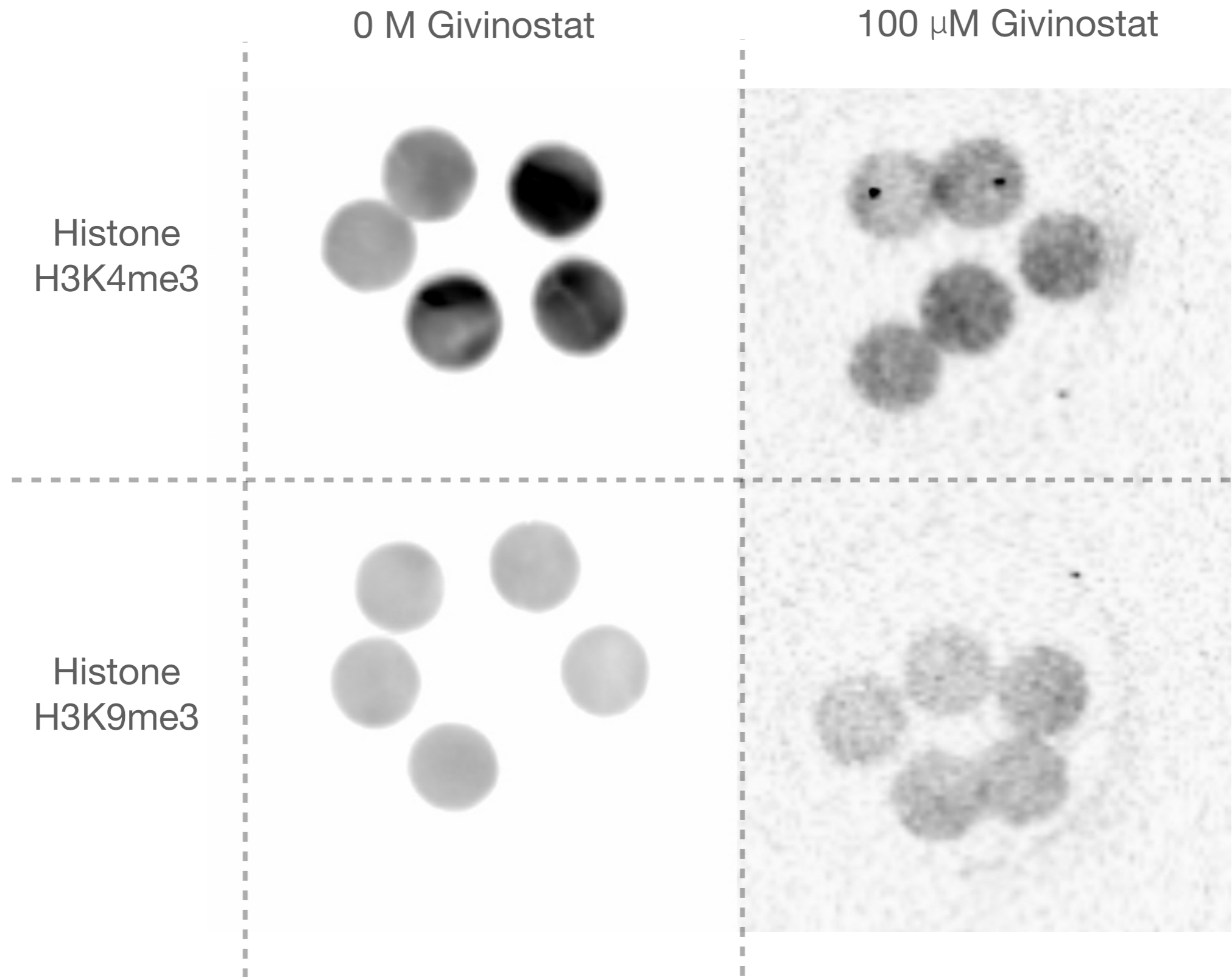

Additional file 10 corresponding to the quantification in Fig 3H  
(chemiluminescence)

Supplement: Supplementary file 10 — Additional file 10. Chemiluminescence scans of DBID blots used for quantification and plotting in Fig. 3h. The Givinostat concentrations and antibodies are indicated along the columns and rows respectively. [file 12885_2020_7526_MOESM10_ESM.pdf]

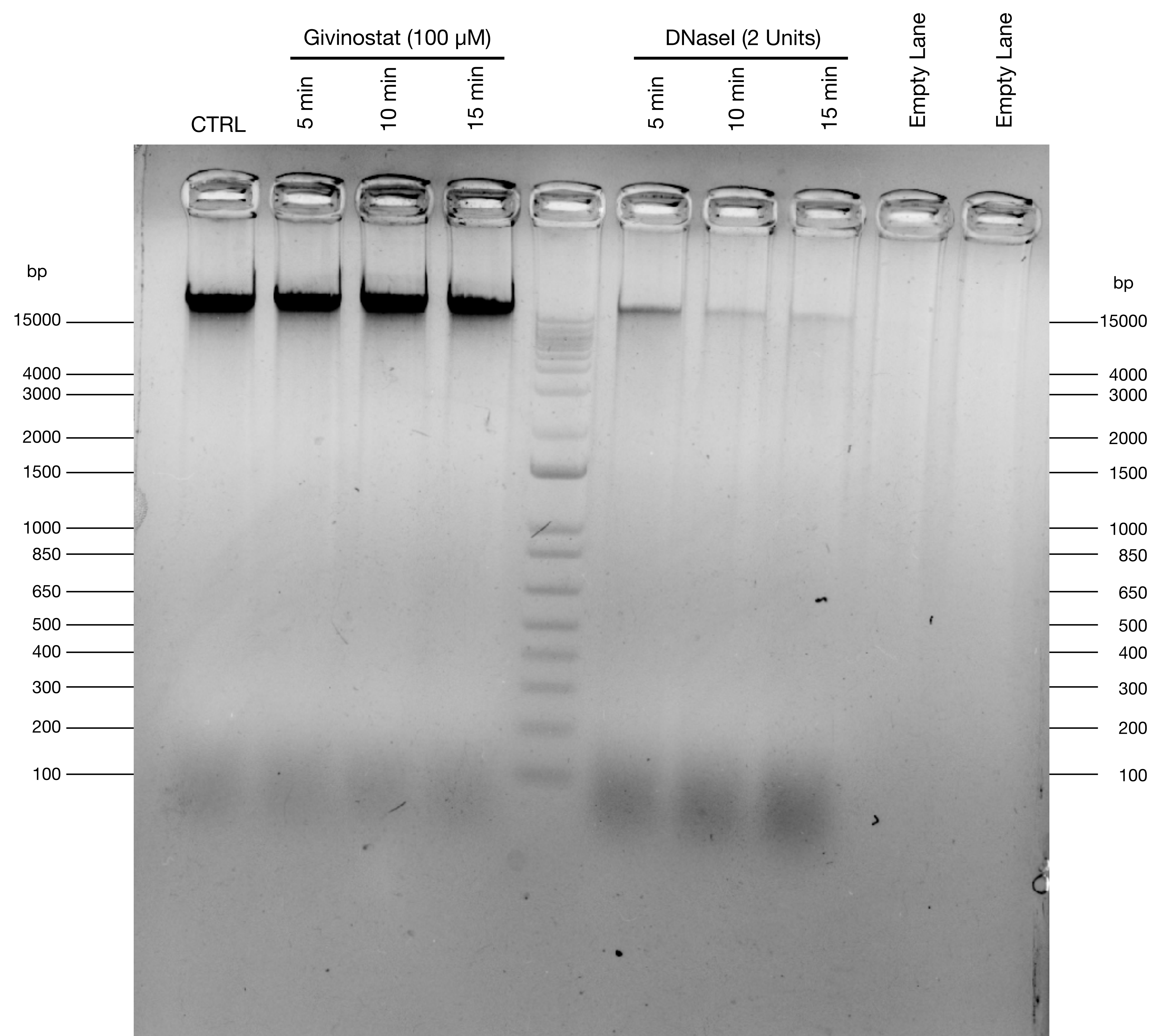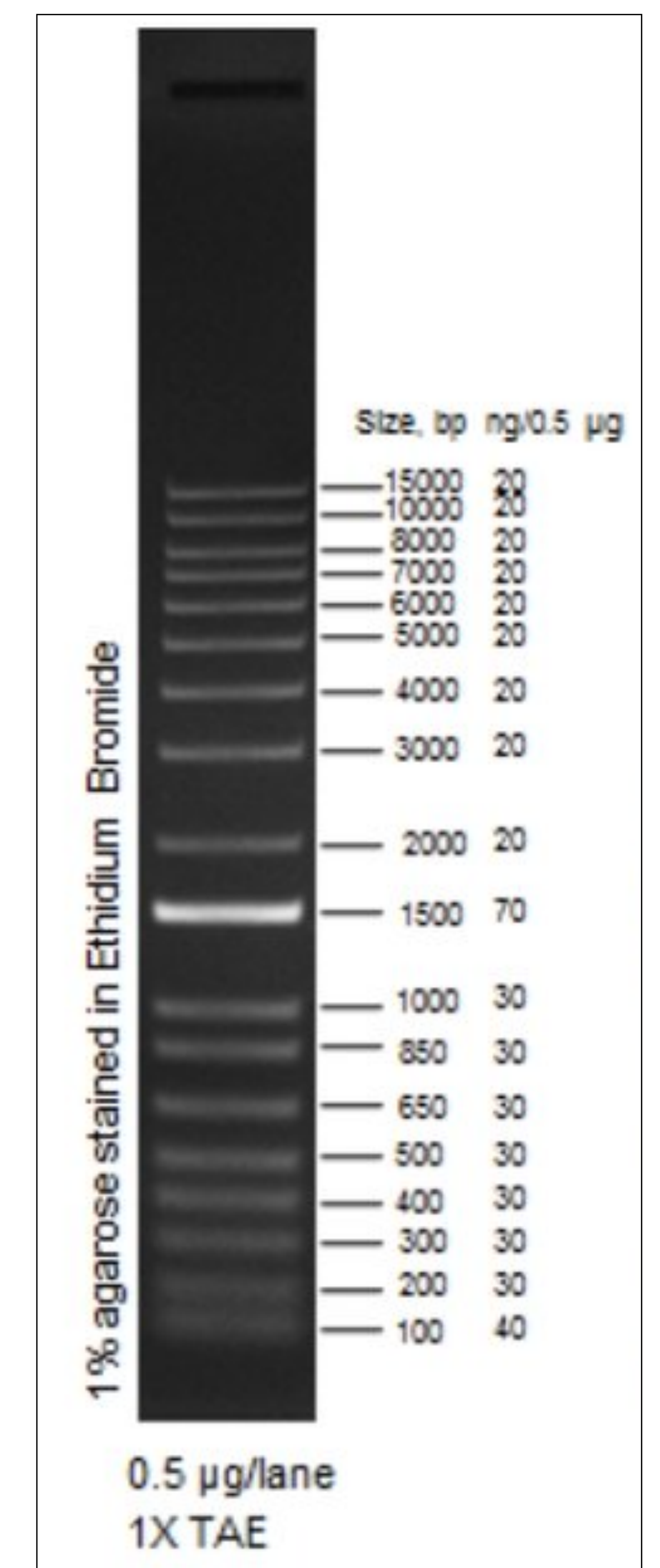

Additional file 13 (corresponding to Fig 3L)

Supplement: Supplementary file 13 — Additional file 13. Raw data for Fig. 3l. Uncropped view of the ethidium bromide-stained agarose gel scan (including the wells and molecular weight marker as indicated) with samples as indicated at the top. The two lanes to the extreme right are empty. [file 12885_2020_7526_MOESM13_ESM.pdf]
