# Supplementary material for: An immunochemistry-based screen for chemical inhibitors of DNA-protein interactions and its application to human CGGBP1
Source: BMC Cancer. 2020 Oct 20;20:1016. doi: 10.1186/s12885-020-07526-5 (PMC7576722; doi:10.1186/s12885-020-07526-5)
Supplement: Supplementary file 2 — Additional file 2. Chemiluminescence scan at a weaker intensity of DBID blots shown in Fig. 3a. The well marked with a red X symbol contains a sample irrelevant to the experiments described. The names of the inhibitors are indicated at the top of each well. [file 12885_2020_7526_MOESM2_ESM.pdf]

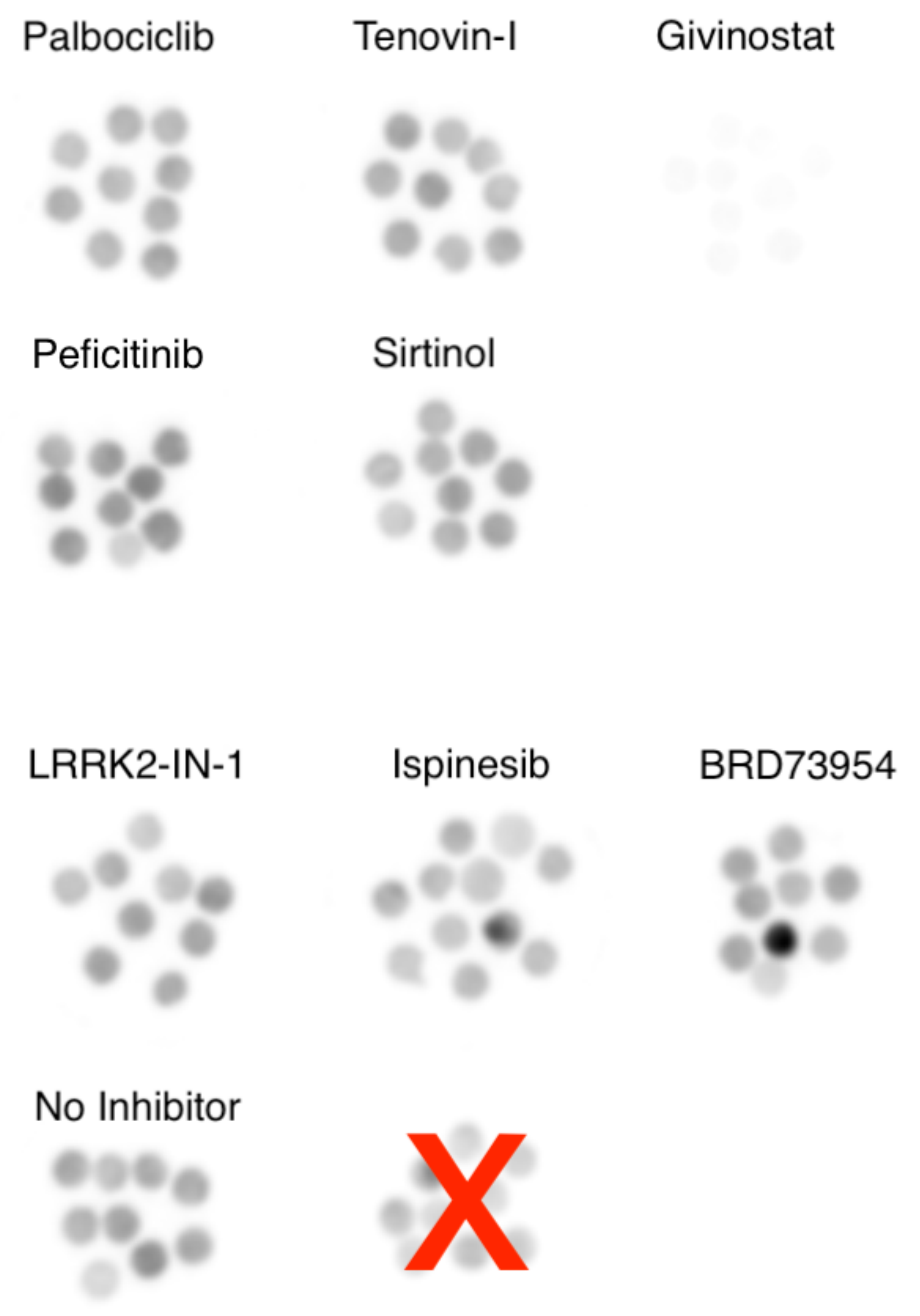

Additional file 2 corresponding to Fig 3A (chemiluminescence, low exposure)
