# Supplementary material for: An immunochemistry-based screen for chemical inhibitors of DNA-protein interactions and its application to human CGGBP1
Source: BMC Cancer. 2020 Oct 20;20:1016. doi: 10.1186/s12885-020-07526-5 (PMC7576722; doi:10.1186/s12885-020-07526-5)
Supplement: Supplementary file 7 — Additional file 7. Chemiluminescence scan of DBID blots used for quantification and plotting in Fig. 3d. The Givinostat concentrations are indicated at the top of each well. [file 12885_2020_7526_MOESM7_ESM.pdf]

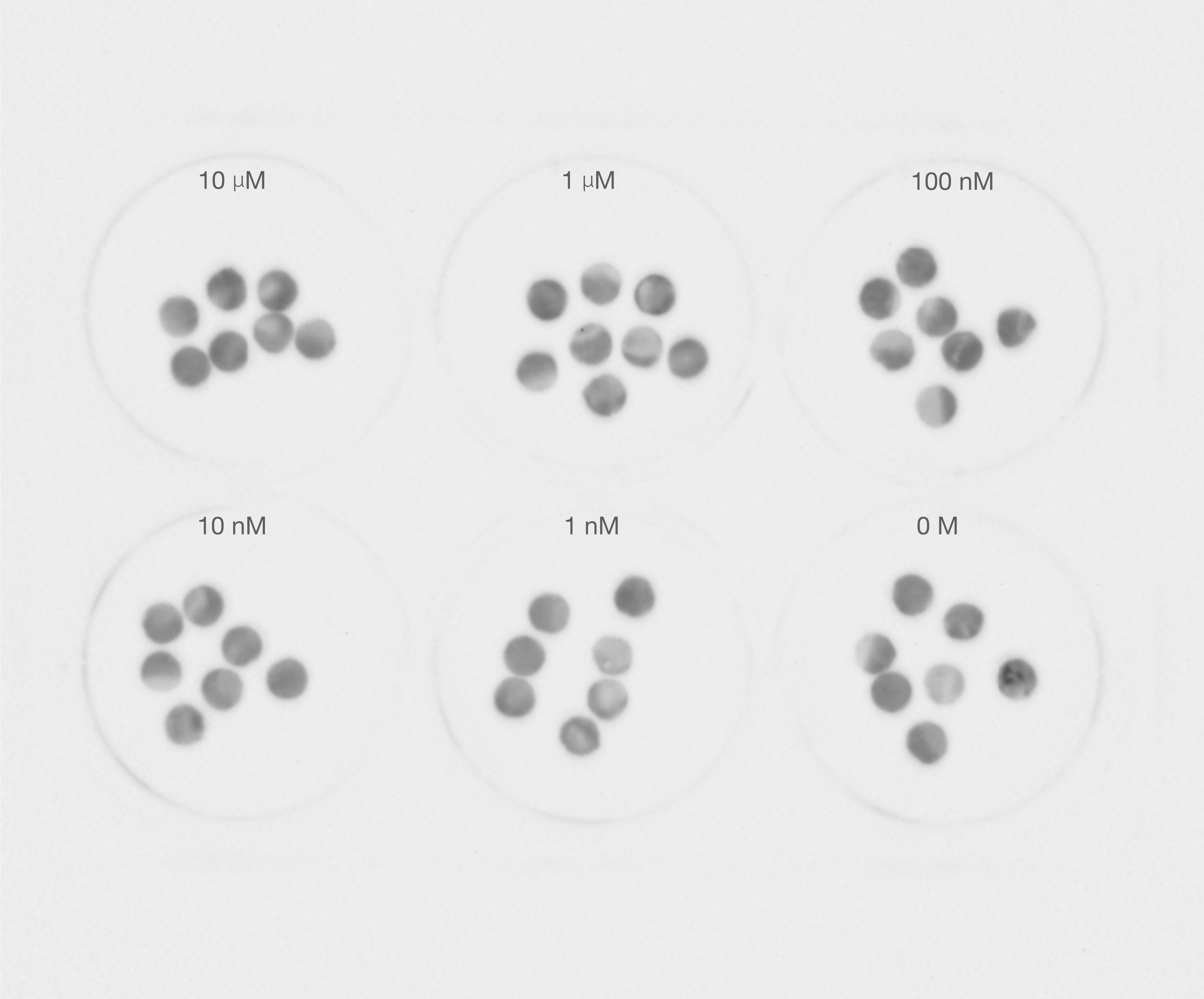

100 µM

Same as the Givinostat sample in Fig 3B

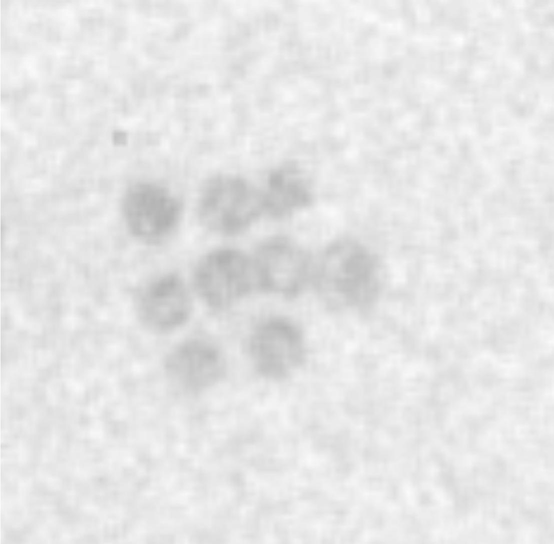

Additional file 7 corresponding to Fig 3D (chemiluminescence, used for quantification)
