# Supplementary material for: An immunochemistry-based screen for chemical inhibitors of DNA-protein interactions and its application to human CGGBP1
Source: BMC Cancer. 2020 Oct 20;20:1016. doi: 10.1186/s12885-020-07526-5 (PMC7576722; doi:10.1186/s12885-020-07526-5)
Supplement: Supplementary file 8 — Additional file 8. Chemiluminescence scans of DBID blots shown in Fig. 3g. The well marked with a red X symbol contains a sample irrelevant to the experiments described. The antibodies used are indicated. [file 12885_2020_7526_MOESM8_ESM.pdf]

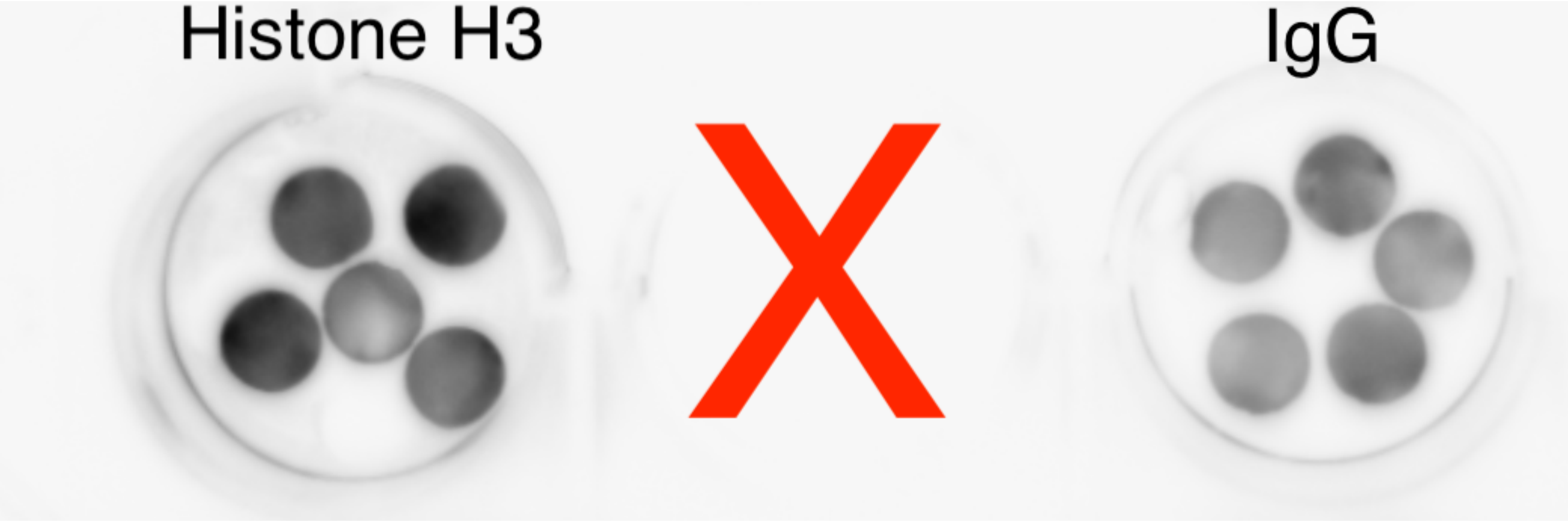

Additional file 8 corresponding to Fig 3G and quantification in Fig 3H (chemiluminescence)
