# Supplementary material for: An immunochemistry-based screen for chemical inhibitors of DNA-protein interactions and its application to human CGGBP1
Source: BMC Cancer. 2020 Oct 20;20:1016. doi: 10.1186/s12885-020-07526-5 (PMC7576722; doi:10.1186/s12885-020-07526-5)
Supplement: Supplementary file 11 — Additional file 11. Raw data for Fig. 3k. A white light scan of the nuclear and cytoplasmic fractionation blot shows the molecular weight marker to the left. A dotted line between 26 kDa and 34 kDa bands indicates the line of incision at which the membrane was divided into two parts. As indicated, the top and the bottom parts were separately probed for GAPDH and CGGBP1 respectively. As indicated, the chemiluminescence scans of CGGBP1 and GAPDH blots are shown either alone or as an overlay with the white light image that shows the molecular weight markers. [file 12885_2020_7526_MOESM11_ESM.pdf]

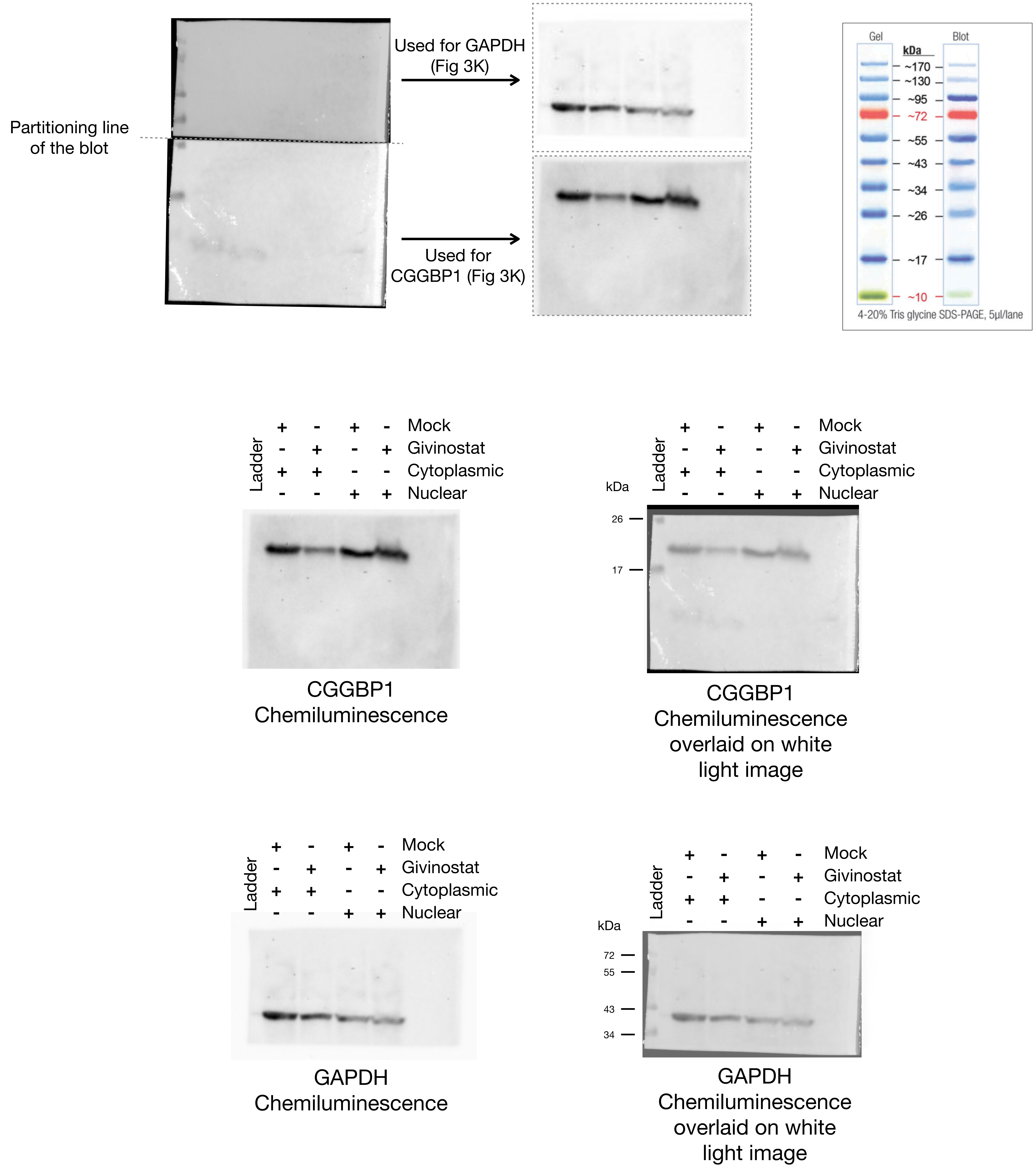

Additional file 11 (corresponding to Fig 3K)
