# Supplementary material for: An immunochemistry-based screen for chemical inhibitors of DNA-protein interactions and its application to human CGGBP1
Source: BMC Cancer. 2020 Oct 20;20:1016. doi: 10.1186/s12885-020-07526-5 (PMC7576722; doi:10.1186/s12885-020-07526-5)
Supplement: Supplementary file 12 — Additional file 12. Raw data for Fig. 3k. As indicated, the chemiluminescence scans of H3K4me3 and total histone H3 blots are shown either alone or as an overlay with the white light image that shows the molecular weight markers. [file 12885_2020_7526_MOESM12_ESM.pdf]

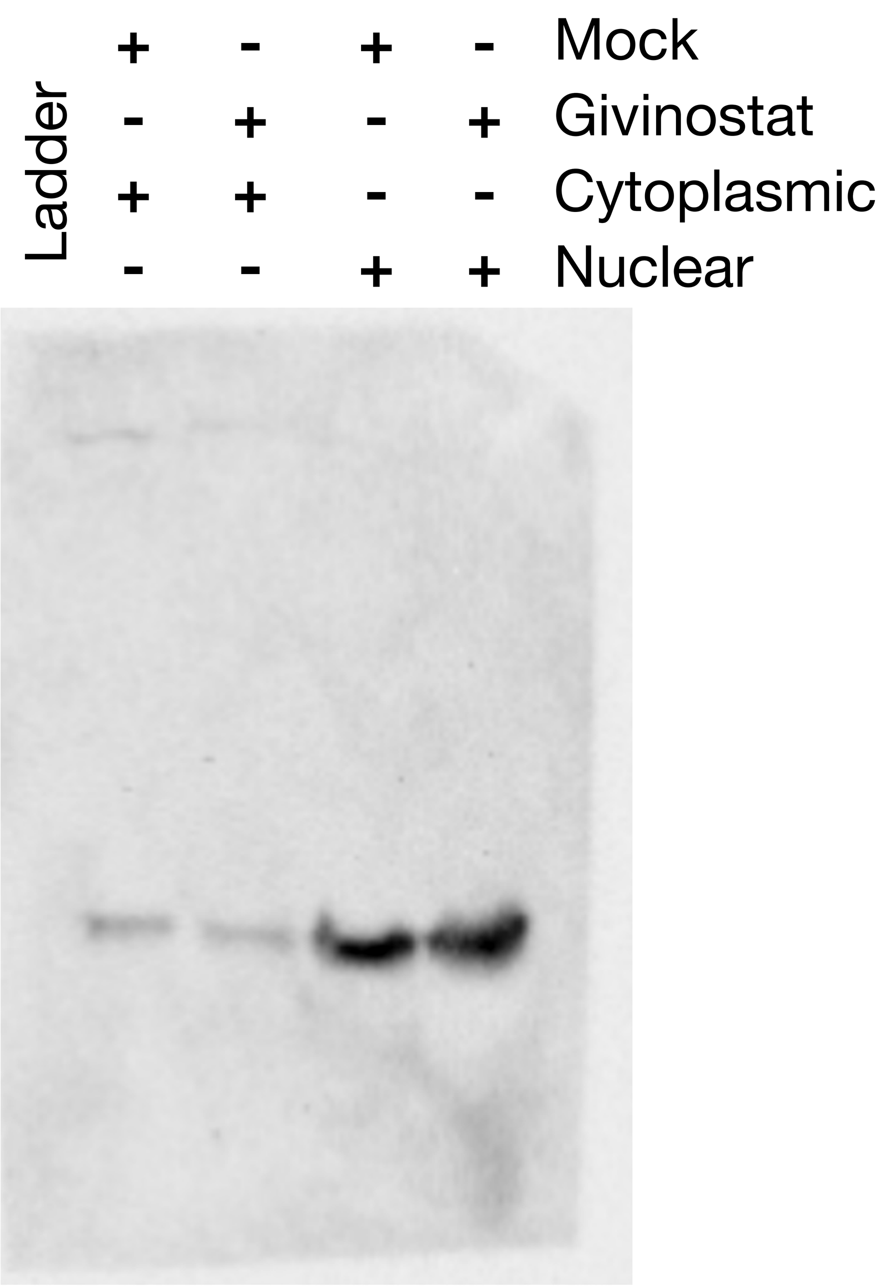

Histone H3K4me3
Chemiluminescence

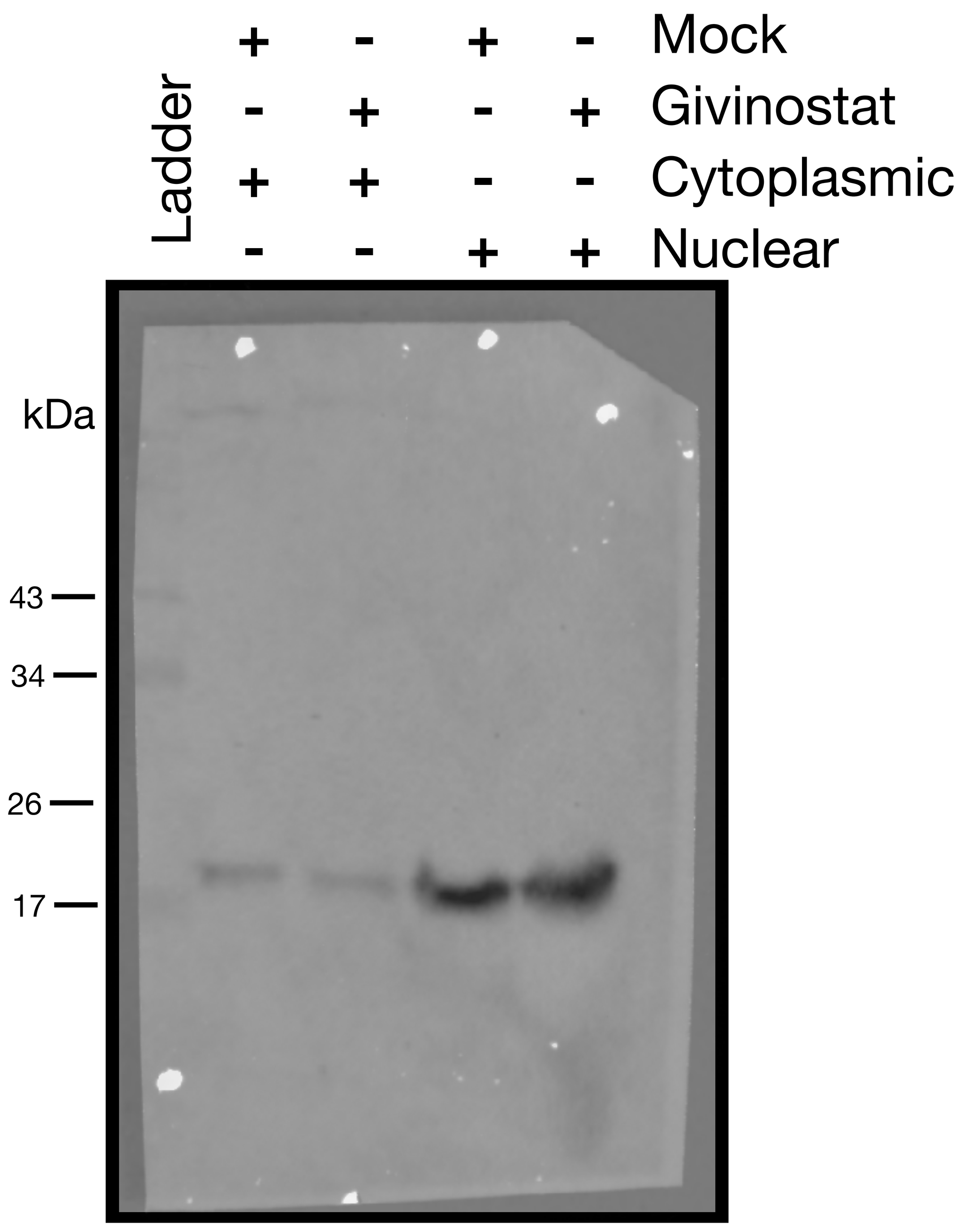

Histone H3K4me3
Chemiluminescence
overlaid on white
light image

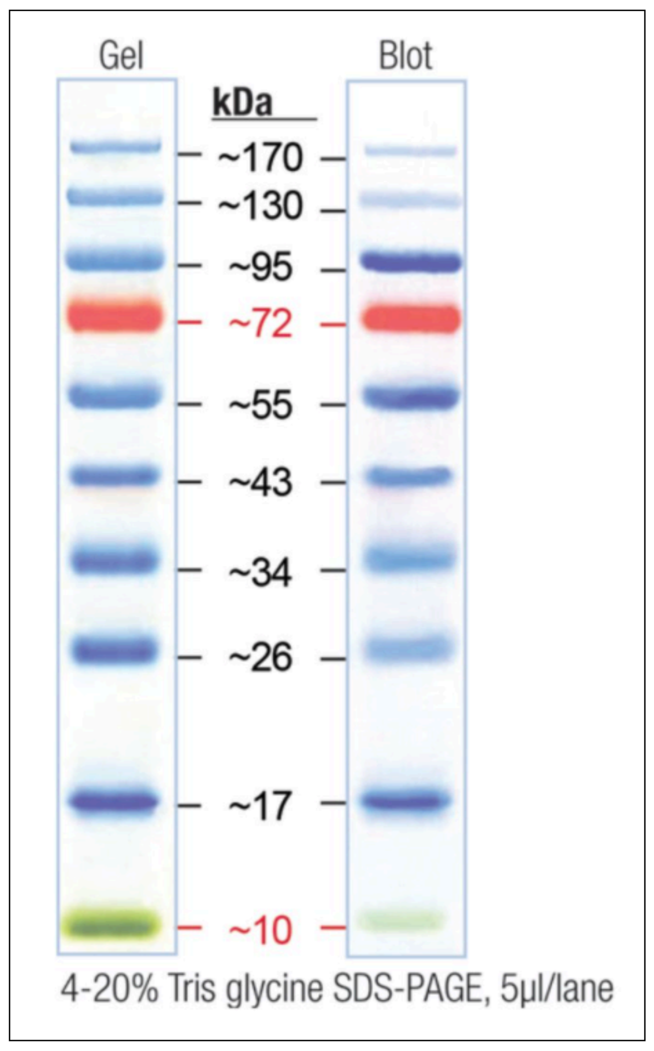

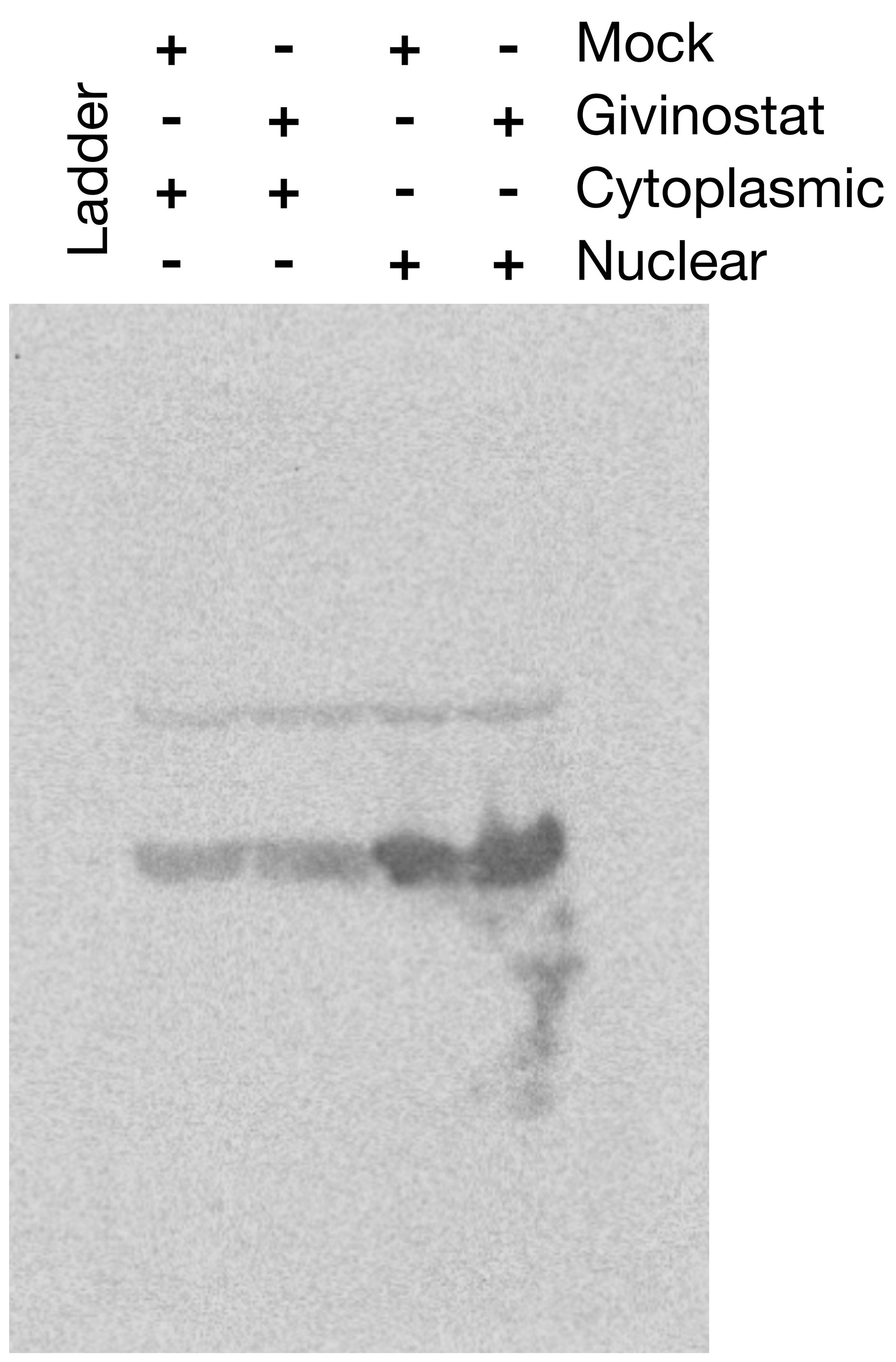

Histone H3
Chemiluminescence

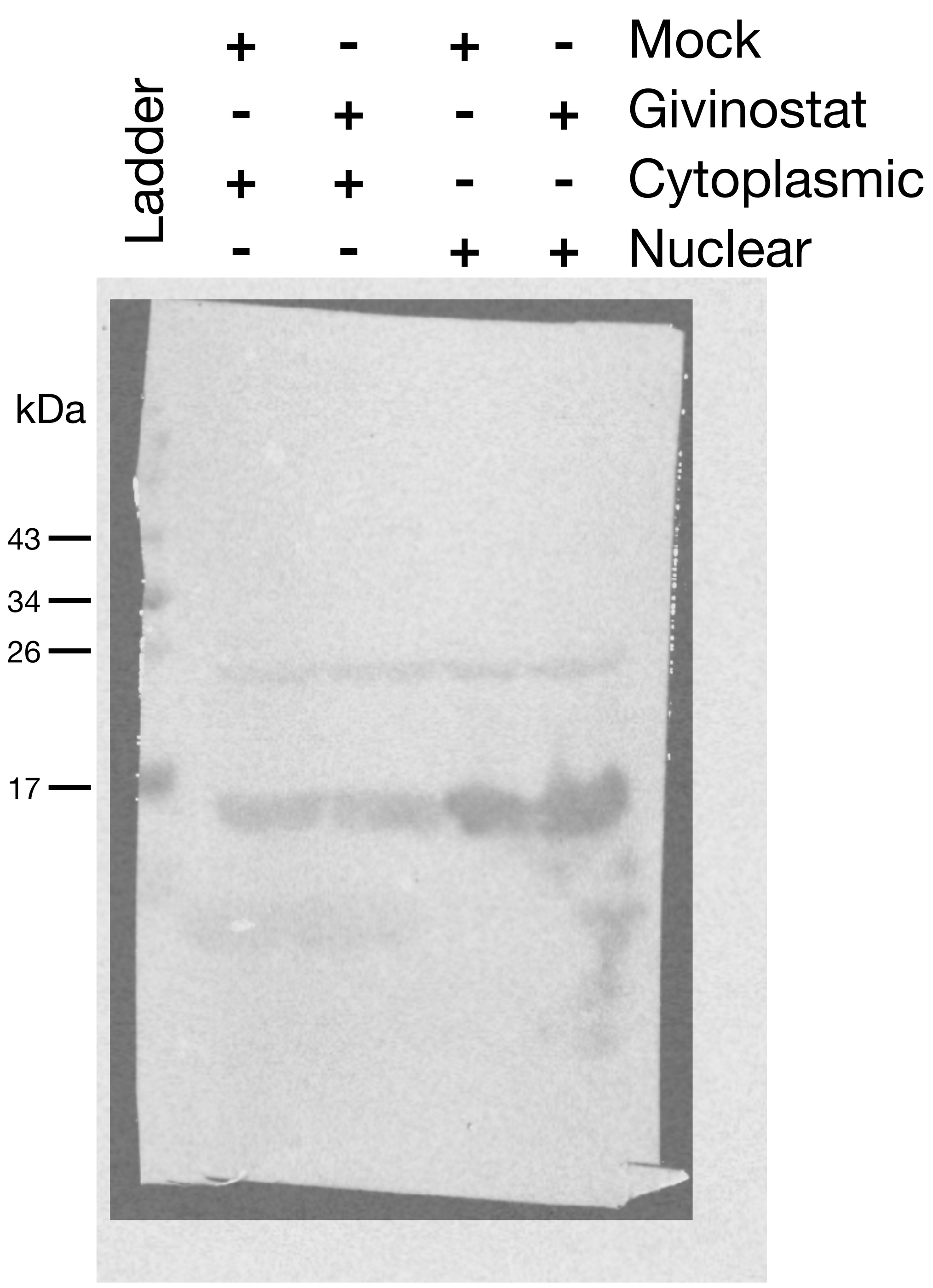

Histone H3
Chemiluminescence
overlaid on white
light image

Additional file 12 (corresponding to Fig 3K)
